# Steroid Phenotype Stratification Reveals Distinct HLA Expression Signatures in Adrenocortical Carcinoma

**DOI:** 10.3390/cancers18020229

**Published:** 2026-01-12

**Authors:** Igor S. Giner, Jean S. S. Resende, João C. D. Muzzi, José A. M. Barbuto, Enzo Lalli, Mauro A. A. Castro, Bonald C. Figueiredo

**Affiliations:** 1 Instituto de Pesquisa Pelé Pequeno Príncipe, Oncology Division, Curitiba 80250-060, PR, Brazil; igor.giner@aluno.fpp.edu.br (I.S.G.); jean.resende@professor.fpp.edu.br (J.S.S.R.); jmuzzi@quantumds.tech (J.C.D.M.); 2 Faculdades Pequeno Príncipe, Pelé Pequeno Príncipe Research Hospital, Oncology Laboratory, Av. Iguaçu, 333-Rebouças, Curitiba 80230-020, PR, Brazil; 3 Bioinformatics and Systems Biology Laboratory, Federal University of Paraná, Curitiba 81520-260, PR, Brazil; 4 Department of Immunology, Institute of Biomedical Sciences, University of São Paulo, São Paulo 05508-000, SP, Brazil; jbarbuto@icb.usp.br; 5 Laboratory of Medical Investigation in Pathogenesis and Targeted Therapy in Onco-Immuno-Hematology (LIM-31), Department of Hematology, Hospital das Clínicas HCFMUSP, Faculty of Medicine, University of São Paulo, São Paulo 05403-000, SP, Brazil; 6 Institut de Pharmacologie Moléculaire et Cellulaire CNRS UMR7275, Inserm U1323, Université Côte d’Azur, 06560 Valbonne, France; lalli@ipmc.cnrs.fr

**Keywords:** Adrenocortical Carcinoma, HLA, steroid phenotype, tumor microenvironment, immune escape, antigen presentation, immune checkpoint blockade, prognosis, biomarker, immunoinformatics

## Abstract

**Background**: Adrenocortical carcinoma (ACC) is a rare, aggressive malignancy where endogenous steroid excess may foster immune evasion. However, whether this hormonal axis directly modulates the antigen presentation machinery remains unclear. **Methods**: We applied an immunoinformatics approach to the TCGA-ACC cohort (*n* = 79) to investigate relationships among steroid phenotype, HLA expression, tumor microenvironment (TME), and patient outcome. Key findings were assessed in an independent validation cohort (ENSAT-ACC, *n* = 44) using C1A/C1B molecular subtypes corresponding to the steroid phenotypes. **Results**: Stratification by steroid phenotype revealed two distinct immunological profiles. The high steroid production (HSP) phenotype was associated with suppressed HLA expression and a lymphocyte-depleted “cold” TME. In contrast, the low steroid production (LSP) phenotype displayed elevated HLA expression, enriched T-cell infiltration, and upregulation of immune checkpoints (e.g., PDCD1, CTLA4), consistent with an inflamed but exhausted TME. The core signature of HLA downregulation in the HSP-like phenotype (C1A) and the significant survival advantage of the LSP-like phenotype (C1B) were confirmed in the validation cohort, demonstrating biological robustness despite platform and sample size differences. **Conclusions**: These findings identify the steroid phenotype as a critical regulator of immune escape in ACC. Our results support incorporating this stratification as a biomarker for patient selection, identifying LSP tumors as the subgroup most likely to benefit from immune checkpoint blockade due to their “hot” yet exhausted microenvironment.

## Simple Summary

Adrenocortical carcinoma is a rare and aggressive cancer often characterized by excess steroid hormone production. We used computational methods to analyze molecular data from patients to investigate whether steroids influence how the immune system recognizes the tumor. Our analysis suggests that tumors stratified by steroid production levels interact with the immune system differently. Tumors producing high levels of steroids show lower levels of the genes that help the immune system recognize cancer, potentially facilitating evasion of the body’s defenses. In contrast, tumors with low steroid production maintain high levels of these genes and are associated with increased immune cell infiltration. This analysis may help explain why patients with high steroid production often have worse outcomes. Our findings suggest that patients with low steroid production might be better candidates for immunotherapies, highlighting the potential of hormone profiles to guide personalized cancer treatment.

## 1. Introduction

Adrenocortical carcinoma (ACC) is a rare yet highly aggressive endocrine neoplasm in adults, with an annual incidence of approximately 2 cases per million people [1]. Despite its rarity, ACC is associated with a poor prognosis, especially in advanced stages, where the five-year survival rate is less than 30% [2,3,4,5,6]. Treatment options for the disease remain limited, with surgical resection as the only curative modality. This underscores the urgent need for a better understanding of the mechanisms driving disease progression and to identify novel therapeutic targets [3,7]

A hallmark of ACC is the frequent overproduction of steroid hormones. While hypercortisolism (excess cortisol) is the most common presentation in adult ACC [8], excessive secretion of androgens, mineralocorticoids, or mixed steroid profiles also occurs [9,10,11]. This hormonal heterogeneity has led to the molecular stratification of ACC into high (HSP) and low (LSP) steroid production subgroups, which have been associated with distinct molecular features and clinical outcomes [8,12].

While immunotherapy has revolutionized the treatment of many solid tumors by leveraging the immune system to eliminate cancer cells, its success relies heavily on antigen presentation. A cornerstone of this recognition process is the presentation of tumor peptides to T cells via Human Leukocyte Antigen (HLA) Class I and II molecules. However, many tumors evade immune surveillance by downregulating HLA expression, thereby rendering malignant cells “invisible” to cytotoxic T lymphocytes [13,14,15].

The interplay between endogenous steroid production by ACC and its immune microenvironment remains a largely unexplored yet promising area of research. Glucocorticoids, such as cortisol, are known for their potent immunosuppressive effects, but their direct impact on the tumor’s ability to be recognized by the immune system has not been fully elucidated in the context of ACC [16,17,18,19]. Notably, a recent pan-cancer analysis by Schaafsma et al. (2021) [20] included ACC but treated it as a homogeneous entity, overlooking potential differences related to steroid phenotype. This raises key unanswered questions: Does the steroid phenotype in ACC modulate HLA gene expression? And consequently, does it determine whether the tumor microenvironment will promote immune escape or productive response against it? To address these questions, our study characterized the HLA expression profiles of ACC stratified by steroid phenotype and evaluated their potential associations with the immune microenvironment and patient outcomes. We conducted an immunoinformatics analysis of The Cancer Genome Atlas (TCGA) cohorts, quantifying class I and II HLA transcripts in HSP versus LSP tumors, relating HLA levels to immune cell infiltration, and testing associations with patient prognosis. We found that LSP tumors are characterized by higher HLA expression, enriched immune infiltration, better survival and co-expression of exhaustion markers, a profile that is typically responsive to immune checkpoint inhibitor (ICI) therapy. Finally, we quantified class I and II HLA transcripts and tested their prognostic value in the independent European Network for the Study of Adrenal Tumors (ENSAT) validation cohort [8,21].

## 2. Materials and Methods

### 2.1. Data Acquisition and Pre-Processing

Transcriptomic and clinical data used in this study were obtained from publicly available cohorts associated with The Cancer Genome Atlas (TCGA) project (accessed on 2 June 2025 via the UCSC Xena Browser, https://xenabrowser.net/datapages/ [22]) and the European Network for the Study of Adrenal Tumors (ENSAT) (accessed on 2 June 2025 via the Gene Expression Omnibus, GEO, https://www.ncbi.nlm.nih.gov/geo/). Three complementary datasets were utilized to address distinct analytical scopes.

For a comparative, large-scale analysis, we used the TCGA Pan-Cancer (PANCAN) dataset [23]. Clinical and survival data (dataset ID: Survival_SupplementalTable_S1_20171025_xena_sp) were filtered to include 10,286 primary tumor samples (TCGA sample type codes “01” to “09”), excluding metastatic and normal tissues. Batch-corrected normalized gene expression data were used (dataset ID: EB++AdjustPANCAN_IlluminaHiSeq_RNASeqV2.geneExp.xena), reported as log_2_(normalized value + 1), which was crucial for enabling robust comparisons across the 33 cancer types.

For focused analyses, we used the TCGA-ACC cohort [8], which includes 79 tumor samples with available RNA-Seq data. Clinical and survival data that were obtained from the files TCGA-ACC.clinical.tsv and TCGA-ACC.survival.tsv.

Of these, the 78 samples with available steroid phenotype information were stratified into high (HSP, *n* = 47) and low (LSP, *n* = 31) steroid production subgroups based on the transcriptional profile classification established by Zheng et al. (2016) [8] and further characterized by Muzzi et al. (2021) [12]. Although clinical hormone data were available (Table 1), transcriptomic stratification was chosen, as it may more accurately reflect the intratumoral steroidogenic machinery [24]. Recent studies have established this cohort as a valuable resource for dissecting ACC heterogeneity [17,25,26,27,28]. Additional annotations for cortisol excess, C1A/C1B molecular subtypes, tumor stage, vital status, and tumor purity were also retrieved. To further characterize the tumor immune landscape, we integrated data from Thorsson et al. (2018) [25], including immune subtypes, stromal fraction, leukocyte fraction, and lymphocyte infiltration scores. Log_2_-transformed Transcripts Per Million (TPM) gene expression data [log_2_(TPM + 1)] were used (dataset ID: TCGA-ACC.star_tpm.tsv). The expression analysis focused on a panel of 22 target genes: 21 HLA genes (Class I and Class II) and B2M gene (which encodes β2-microglobulin, an essential component of HLA class I molecules). This panel was utilized to maintain consistency with previous pan-cancer HLA studies established by Schaafsma et al. (2021) [20].

To validate key findings in silico, we utilized an independent cohort from the European Network for the Study of Adrenal Tumors (ENSAT) [21], accessed via the GEO portal (accession number GSE49278). This validation dataset comprises 44 ACC tumor samples. Clinical data and the normalized microarray gene expression matrix (Robust Multiarray Average, RMA; Affymetrix platform) were retrieved using the GEOquery R package (v.2.72.0) [29]. Ambiguous probes were excluded during pre-processing. Consequently, HLA-DQA2 was unavailable for this cohort, reducing the analyzed panel to 21 genes (20 HLA genes and B2M) for the validation steps. For genes with multiple probes, the one with the highest coefficient of variation across samples was selected to represent the gene expression. Since steroid phenotype data were unavailable for this cohort, samples were stratified based on the C1A/C1B molecular subtypes, which present a high overlap with HSP and LSP phenotypes, respectively, as established in the literature [30]. Additional clinical annotations were obtained from the supplementary tables of [21].

### 2.2. Analysis of HLA Gene Expression in Pan-Cancer

To contextualize HLA expression in ACC within the pan-cancer setting, two sequential comparative analyses were performed. We first established a baseline profile of HLA expression [log_2_(normalized value + 1)] by calculating the median expression of 22 genes (21 HLA genes plus B2M) across each of the 33 cancer types represented in the PANCAN cohort. In this step, ACC samples were analyzed as a single, unified group (*n* = 79). In a second analysis to investigate ACC heterogeneity, the cohort was reconfigured: the ACC samples were stratified by steroid phenotype, and their subgroups, HSP (*n* = 47) and LSP (*n* = 31), were treated as independent entities alongside the 32 remaining cancer types, totaling 34 comparative groups. To visualize the resulting expression patterns, heatmaps were generated using the pheatmap R package (v1.0.12) [31], with unsupervised hierarchical clustering to arrange genes and cancer types based on expression similarity. Additionally, the expression distribution of a subset of three genes of interest (HLA-DPA1, HLA-DPB1, and HLA-DRA) was visualized using individual boxplots, with cancer types ordered by ascending median expression.

### 2.3. Calculation of the HLA Pathway Signature Score

We derived a single HLA pathway signature as follows. For each gene in a 21-gene HLA panel (excluding B2M), expression was z-standardized gene-wise across the full pan-cancer dataset. A sample-level HLA score was then computed as the arithmetic mean of the 21 z-scores. For each cancer type (cohort), we obtained a cohort-level score by averaging the sample-level scores. Cancer types were ranked by the cohort-level score and visualized in a dot plot.

### 2.4. Analysis of HLA Gene Expression in Adrenocortical Carcinoma

To investigate the association between steroid phenotype and HLA expression, we compared the expression [log_2_(TPM + 1)] of 22 target genes (21 HLA genes and B2M) between the HSP (*n* = 47) and LSP (*n* = 31) subgroups of the TCGA-ACC cohort. The Mann–Whitney U test was used for this comparison, and a Benjamini–Hochberg adjusted *p*-value < 0.05 was considered statistically significant. To validate these results, the same differential expression analysis was performed on the ENSAT-ACC cohort, comparing HLA/B2M expression (RMA-normalized) between the C1A (HSP-like) and C1B (LSP-like) molecular subtypes (20 HLA genes and B2M).

To visualize the expression patterns and their relationship with the clinicopathological and immunological characteristics of the samples, integrative heatmaps were generated using the ComplexHeatmap R package (v2.20.0) [32]. To prepare the expression data for the visualization, the values for each gene were standardized by z-score scaling across all ACC samples. Subsequently, to mitigate the effect of extreme values on the color scale and improve visualization, the z-scores were rescaled to a range of −2 to 2. Samples (columns) in the heatmap were ordered by unsupervised hierarchical clustering, based exclusively on their 22 HLA/B2M gene expression profiles.

### 2.5. Unsupervised Hierarchical Clustering

To assess natural sample grouping, unsupervised hierarchical clustering of the TCGA-ACC samples (*n* = 78) was performed based on HLA/B2M expression. Prior to analysis, gene expression values were standardized by z-score scaling across all samples. The clustering utilized Euclidean distance and the complete linkage method. The resulting groups were visualized using the ComplexHeatmap R package (v2.20.0) [32], with annotations for steroid phenotype, cortisol excess, and molecular subtype.

### 2.6. Analysis of Immune Cell Infiltration

The abundance of immune cell populations within the tumor microenvironment (TME) of the TCGA-ACC cohort was estimated from gene expression data using the MCP-counter method [33], implemented via the IOBR R package (v0.99.0) [34]. The resulting abundance scores were used in the TCGA cohort for correlation analyses with steroid phenotypes and HLA gene expression. To assess the consistency of the immune microenvironment profile in an independent setting, the abundance of immune cell populations in the ENSAT-ACC cohort was also estimated using the same methodology.

### 2.7. Analysis of T/B Cell and Exhaustion Markers

To investigate functional relationships between the HLA pathway and the immune context, we correlated the expression of HLA/B2M genes with a panel of genes representing various immune phenotypes (TPM values). This panel included markers for T and B cells curated from Abcam (available at https://www.abcam.com/en-us/technical-resources/research-areas/marker-guides/t-cell-markers?srsltid=AfmBOopayrroStCkLfEyKK91jGbYVjN1iCcqIsVIOaP-4QjbHC9eaaYY and https://www.abcam.com/en-us/technical-resources/research-areas/marker-guides/b-cell-markers, accessed on 30 June 2025), and immune checkpoint genes associated with immune evasion and exhaustion established by Acharya et al. (2020) [35]. For visualization, a heatmap was generated using the ComplexHeatmap R package (v2.20.0) [32]. In the heatmap, samples (columns) were ordered by unsupervised hierarchical clustering based exclusively on their 22 HLA/B2M gene expression profiles.

### 2.8. Correlation of HLA Gene Expression with Immune Cell Infiltration, and with Markers of T/B Cells and Exhaustion

Correlation analyses were performed separately for the HSP and LSP subgroups using Spearman’s rank correlation. For each subgroup, a correlation matrix was generated, and the resulting *p*-values were adjusted for multiple comparisons using the Benjamini–Hochberg method to control the False Discovery Rate (FDR). Associations with an FDR < 0.05 were considered statistically significant. The results were visualized as correlation heatmaps generated with the corrplot R package (v0.95) [36].

### 2.9. Survival Analysis

To evaluate the prognostic impact of steroid phenotypes, C1A/C1B molecular subtypes, and HLA expression, overall survival (OS) analyses were performed in both the TCGA-ACC and ENSAT-ACC cohorts. Survival curves were generated using the Kaplan–Meier method, and differences between groups were assessed using the log-rank test. In the TCGA-ACC cohort, patients were stratified by steroid phenotype (HSP versus LSP), molecular subtypes (C1A versus C1B), tumor stage, individual HLA/B2M gene expression levels (dichotomized as ‘high’ or ‘low’ based on the median), and the HLA pathway signature score. Similarly, for the ENSAT-ACC validation cohort, survival comparisons were performed based on the molecular subtypes C1A (HSP-like) and C1B (LSP-like), tumor stage, and individual HLA gene expression levels.

To determine whether HLA/B2M expression was an independent prognostic factor in the TCGA-ACC cohort, a multivariate Cox proportional hazards model was fitted. In this model, individual HLA/B2M expression or the HLA pathway signature score was assessed as the primary variable, after adjusting for tumor stage and the steroid phenotype (clinically relevant confounding covariates). All survival analyses and visualizations were performed in R using the survival (v. 3.7.0) [37] and survminer (v. 0.5.0) [38] packages.

### 2.10. Statistical Analysis

All statistical analyses were performed in R (v. 4.4.1). Two-group comparisons were performed as follows: for continuous variables with a non-normal distribution (as determined by the Shapiro–Wilk test), the Mann–Whitney U test was used [39,40], while categorical variables were analyzed using Fisher’s exact test [41]. A significance level of *p* < 0.05 was considered. For analyses involving multiple comparisons, the Benjamini–Hochberg method [42] was applied to control the false discovery rate (FDR < 0.05).

## 3. Results

### 3.1. Study Design

**Figure 1:**
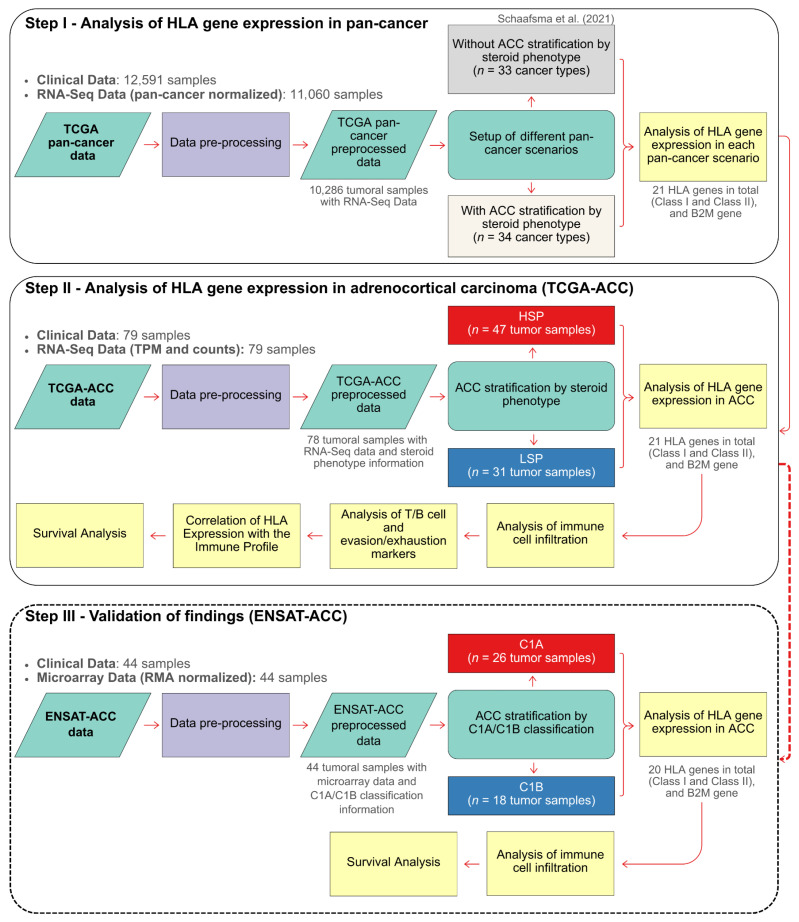
Flowchart of the study design. The analytical strategy was divided into three main steps. Step I: Analysis of HLA/B2M Gene Expression in Pan-Cancer. This step established a reference landscape by comparing a scenario with an unstratified ACC cohort (33 cancer types), following Schaafsma et al. (2021) [20], to one that distinguishes the ACC HSP and LSP subgroups (totaling 34 cancer types). Step II: In-Depth Analysis of the TCGA-ACC Cohort. This step investigated the direct differences in HLA/B2M expression between the HSP and LSP phenotypes and their association with immune cell infiltration, T/B cell and evasion/exhaustion markers, and patient survival. Step III: Validation of Findings (ENSAT Cohort). This final step aimed to validate the findings from the TCGA-ACC cohort by analyzing HLA/B2M gene expression in the independent ENSAT-ACC cohort, which was stratified into the C1A and C1B molecular subtypes. This also included an analysis of immune cell infiltration and patient survival within the ENSAT cohort.

To test the hypothesis that the steroid phenotype modulates HLA expression in ACC, we adopted a three-step analytical strategy (Figure 1). Step I (Pan-cancer analysis): Expression of HLA and B2M genes was compared across 33 TCGA cancer types (detailed sample counts in Supplementary Table S1) to contextualize ACC within the broader tumor landscape. Analyses were performed first with ACC as a single unstratified group and then stratified into high (HSP) and low (LSP) steroid production subgroups (34 groups in total). Step II (ACC-focused analysis): An in-depth evaluation of the TCGA-ACC cohort was performed to dissect molecular and immunological differences between HSP and LSP tumors, focusing on (i) HLA expression, (ii) immune infiltration, including T/B cell and evasion/exhaustion markers, and (iii) associations with patient survival. Step III (Validation): This final step involved the in silico validation of key findings from the TCGA analysis using the independent ENSAT-ACC cohort [21]. Since steroid phenotype data was unavailable for the ENSAT-ACC cohort, this validation focused on the literature-known, overlapping molecular subtypes, C1A (HSP-like) and C1B (LSP-like) [30].

### 3.2. Clinicopathological and Immunological Characteristics of the HSP and LSP Subgroups

First, we characterized The Cancer Genome Atlas (TCGA) Adrenocortical Carcinoma (ACC) cohort (*n* = 78) based on its stratification into high (HSP) and low (LSP) steroid production phenotypes. The demographic, clinicopathological, and immunological characteristics of each subgroup are detailed in Table 1.

Stratification by steroid phenotype revealed notable differences in several parameters. As expected, cortisol overproduction was predominant in the HSP subgroup (57% of cases) compared to the LSP subgroup (16%).

Consistent with established literature, a C1A/C1B molecular subtypes distinction was observed, whereby the HSP subgroup was primarily composed of the C1A subtype (85% of cases), and the LSP subgroup was largely defined by the C1B subtype (90% of cases) [30,43].

A distinction was also observed in the tumor immune subtypes as defined by Thorsson et al. (2018) [25]. The HSP subgroup was dominated by the C4 (‘Lymphocyte Depleted’) immune subtype, accounting for 79% of its cases. In contrast, the LSP tumors were enriched in the “inflammatory” (C3) subtype (52%). These findings highlight fundamental differences in the immune microenvironment between the two steroid phenotypes.

To provide context for our validation steps, we also characterized the independent ENSAT-ACC cohort, stratified by the C1A and C1B molecular subtypes (Supplementary Table S2). While the association between the HSP-like C1A subtype and aggressive clinical features was preserved, highlighted by the higher prevalence of Stage IV tumors in the high-risk subgroups of both cohorts (38% in ENSAT C1A and 26% in TCGA HSP) compared to their low-risk counterparts (17% in ENSAT C1B and 6% in TCGA LSP), the ENSAT-ACC cohort differs from the TCGA dataset in terms of sample size (*n* = 44 vs. *n* = 78) and demographics. The ENSAT-ACC C1B subgroup presented a higher proportion of female patients (89%) compared to the TCGA-ACC LSP subgroup (52%). These distinctions, particularly the reduced statistical power inherent to the smaller cohort size, provide essential context for interpreting the validation results

### 3.3. Stratification by Steroid Phenotype Redefines the HLA Expression Profile of Adrenocortical Carcinoma in the Pan-Cancer Context

**Figure 2:**
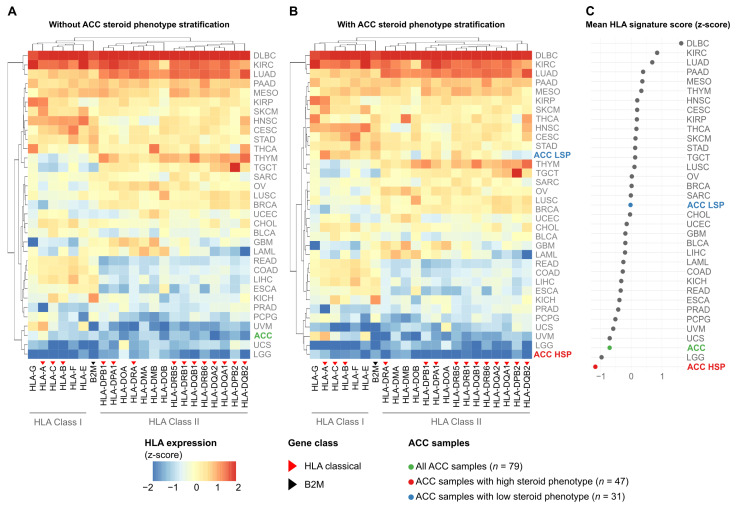
Stratification of ACC by steroid phenotype reveals a distinct HLA expression signature in the Pan-Cancer setting. Heatmaps display the median expression (z-score standardized per gene across all cohorts) of 21 HLA genes and B2M (columns) across 33 TCGA cancer types (rows). Blue indicates low relative expression, and red indicates high relative expression. (**A**) Pan-cancer analysis with ACC (*n* = 79) as a single group. (**B**) Pan-cancer analysis with ACC stratified into HSP (*n* = 47) and LSP (*n* = 31). Rows and columns in (**A**,**B**) are ordered by hierarchical clustering. (**C**) Dot plot showing the mean HLA pathway signature score (mean of the 21 HLA gene z-scores) for each cancer type, ranked from lowest to highest. ACC and its subgroups are highlighted in color.

The initial pan-cancer analysis of median HLA/B2M expression revealed a wide range across 33 cancer types (Figure 2A). While hematological cancers such as Diffuse Large B-cell Lymphoma (DLBC) and Clear Cell Renal Cell Carcinoma (KIRC) exhibited the highest expression levels, ACC, when analyzed as a single group, clustered among the tumors with the lowest expression, alongside Low-Grade Glioma (LGG) and Uveal Melanoma (UVM).

However, this landscape changed upon stratification of the ACC cohort by steroid phenotype, revealing a notable divergence (Figure 2B). The low steroid production subgroup (ACC LSP) repositioned to an intermediate expression level, clustering with cancer types such as Lung Squamous Cell Carcinoma (LUSC) and Breast Cancer (BRCA). In striking contrast, the high steroid production subgroup (ACC HSP) was isolated at the lowest end of the spectrum, exhibiting the lowest HLA/B2M expression among all 34 analyzed cancer types.

To quantify this observation, we calculated a mean HLA pathway signature score for each group (Figure 2C). Although the unstratified ACC cohort already had one of the lowest scores, stratification revealed that this overall low signal was primarily driven by the ACC HSP subgroup, which exhibited the lowest mean HLA pathway signature score of all cohorts. Conversely, the score in the ACC LSP subgroup was markedly higher, ranking near the pan-cancer median.

### 3.4. HLA Class II Genes Linked to Pediatric ACT Show Divergent Expression in Adult ACC

Given prior associations of HLA-DPA1, HLA-DPB1, and HLA-DRA with pediatric ACT (pACT) prognosis [44,45,46], we examined their expression in adult ACC (Figure S1). To investigate whether these genes also exhibit altered expression in adult ACC, we evaluated their profiles within the pan-cancer context.

Our analysis revealed a consistent and notable pattern for all three genes (Figure S1A–C). When analyzed as a single group (green boxes), ACC ranked among the cancer types with the lowest median expression compared to the 32 others. However, stratification by steroid phenotype revealed that the low steroid production subgroup (ACC LSP, blue boxes) exhibited higher expression, repositioning itself to an intermediate rank in the pan-cancer setting. In contrast, the high steroid production subgroup (ACC HSP, red boxes) consistently ranked among the lowest positions, displaying the lowest median expression levels compared to all other cancer types.

### 3.5. The LSP Phenotype Exhibits a Signature of Higher HLA Expression and a “Hot” Tumor Microenvironment

**Figure 3:**
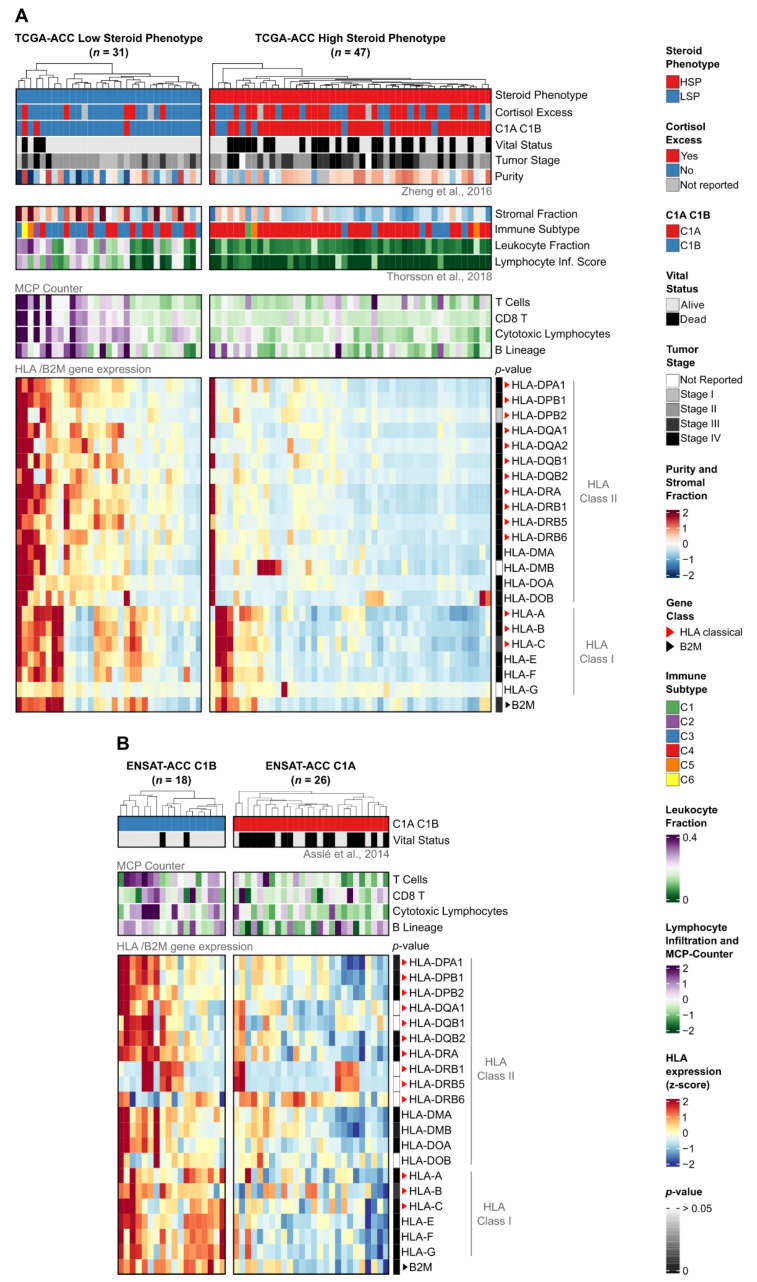
Expression of HLA/B2M genes and association with clinical, compositional, and immunological features in TCGA-ACC and ENSAT-ACC cohorts. Heatmaps of (**A**) TCGA-ACC tumors stratified by steroid phenotype (LSP, *n* = 31; HSP, *n* = 47) and (**B**) ENSAT-ACC tumors stratified by C1A/C1B molecular subtypes (C1B, *n* = 18; C1A, *n* = 26). In both panels, columns represent individual samples hierarchically clustered within subgroups based on HLA/B2M expression. (**Top panels**) display clinical and immunological annotations, with compositional features included for (**A**). (**Bottom panels**) show normalized gene expression z-scores scaled from −2 (blue) to +2 (red), derived from TPM + 1 for (**A**) or RMA-normalized values for (**B**). Sidebars indicate Benjamini–Hochberg adjusted *p*-values (Mann–Whitney U test) for subgroup comparisons (dark gray: low *p*-value; white: *p* > 0.05). Red and black triangles indicate classical HLA genes and the B2M gene, respectively. Clinical and molecular annotations were integrated from Zheng et al. (2016), Thorsson et al. (2018), and Assié et al. (2014) [8,21,25].

Integration of HLA/B2M expression with clinicopathological and immunological features (Figure 3) revealed two distinct biological signatures that align with the steroid phenotype stratification in TCGA and the C1A/C1B molecular subtypes in ENSAT.

Notably, unsupervised hierarchical clustering based exclusively on HLA/B2M gene expression showed that the tumors naturally segregate into clusters that align closely with the established steroid phenotypes and clinical cortisol status (Supplementary Figure S2).

Consistent with the pan-cancer findings, LSP tumors in the TCGA cohort exhibited higher expression levels for the vast majority (19 out of 21) of analyzed HLA genes compared to HSP tumors (Mann–Whitney test, Benjamini–Hochberg adjusted *p*-value < 0.05; Figure 3A, *p*-value sidebar; Supplementary Figure S3). This difference was particularly evident for the Class II genes, such as HLA-DPA1 (*p* = 1.38 × 10^−6^), HLA-DPB1 (*p* = 4.42 × 10^−6^), and HLA-DRA (*p* = 2.63 × 10^−6^). Only HLA-G (*p* = 0.29) and HLA-DMB (*p* = 0.09) did not show statistically significant differences.

To validate these findings, we analyzed the independent ENSAT-ACC cohort stratified by C1A/C1B molecular subtypes (Figure 3B). The C1B subtype (analogous to LSP) displayed a significantly higher HLA expression profile compared to C1A (analogous to HSP). Specifically, 15 out of 21 genes were differentially expressed (Supplementary Figure S4). We compared these results with a C1A/C1B stratification of the TCGA cohort (Supplementary Figure S5) and identified a core set of 12 HLA genes that were consistently upregulated in the low-risk phenotypes (LSP/C1B) across both cohorts (Supplementary Figure S6). Crucially, within the ENSAT cohort, this core set included the pediatric ACT-associated genes HLA-DPA1 (*p* = 5.85 × 10^−3^), HLA-DPB1 (*p* = 3.75 × 10^−3^), and HLA-DRA (*p* = 8.94 × 10^−4^). Although reduced statistical power in the smaller ENSAT-ACC cohort and technical differences between profiling platforms (RNA-Seq versus microarray) limited significance for some genes, the overall pattern of HLA downregulation in the high-risk phenotypes (HSP/C1A) remained consistent.

Beyond gene expression, the stratification revealed distinct microenvironmental profiles. In the TCGA-ACC cohort, LSP tumors were characterized by lower tumor purity, higher stromal and leukocyte fractions, and elevated infiltration scores for T and B lymphocytes (Figure 3A top panels; Supplementary Figure S7A). Supporting this “hot” immune microenvironment, the ENSAT-ACC C1B subtype also exhibited significantly higher infiltration scores for cytotoxic lymphocytes (*p* = 2.65 × 10^−2^) compared to the C1A subtype (Figure 3B top panels; Supplementary Figure S7B).

### 3.6. HLA Expression Associates with T-Cell Infiltrate Only in the LSP Phenotype

**Figure 4:**
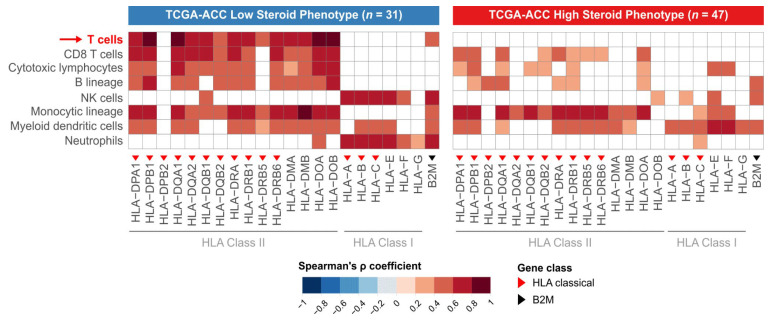
Contrasting patterns of correlation between HLA expression and immune infiltrate in HSP and LSP subgroups. Heatmaps display the Spearman correlation coefficients (ρ) between the expression of individual HLA/B2M genes (x-axis) and the abundance scores of immune cell populations (y-axis), derived from the MCP-counter method [33]. The analysis was performed separately for the LSP (*n* = 31 (**left panel**)) and HSP (*n* = 47 (**right panel**)) subgroups. The color scale indicates the strength of positive correlation, with darker red representing stronger correlations. White tiles indicate non-significant correlations (Benjamini–Hochberg adjusted *p*-value > 0.05). Red triangles denote classical Class I or II HLA genes, and the black triangle denotes the B2M gene. The red arrow highlights the T-cell row to draw attention to a key contrast between the HSP and LSP phenotypes.

Having established that LSP tumors have higher HLA expression and greater lymphocyte infiltration, we next sought to determine whether a correlation exists between these two features. To this end, we performed Spearman correlation analyses between the expression of each HLA/B2M gene and the abundance scores of various immune cells, separately for each steroid phenotype (Figure 4).

In the LSP subgroup, we observed a positive correlation between the expression levels of most HLA Class II genes and the abundance of lymphocyte populations. This pattern is indicative of an immunologically active tumor microenvironment, where high HLA expression is possibly functionally coupled to the recruitment and presence of effector T and B cells.

In contrast, the association between HLA Class II gene expression and the abundance of lymphocyte populations was lost in the HSP subgroup. In this phenotype, no significant correlation was observed between HLA gene expression and T-cell abundance. This loss of correlation suggests an inactive antigen presentation pathway, a feature characteristic of an immunosuppressed tumor microenvironment where the presence of HLA on tumor cells is not sufficient to ensure lymphocyte infiltration.

### 3.7. LSP Microenvironment Shows Simultaneous T-Cell Activation and Exhaustion

**Figure 5:**
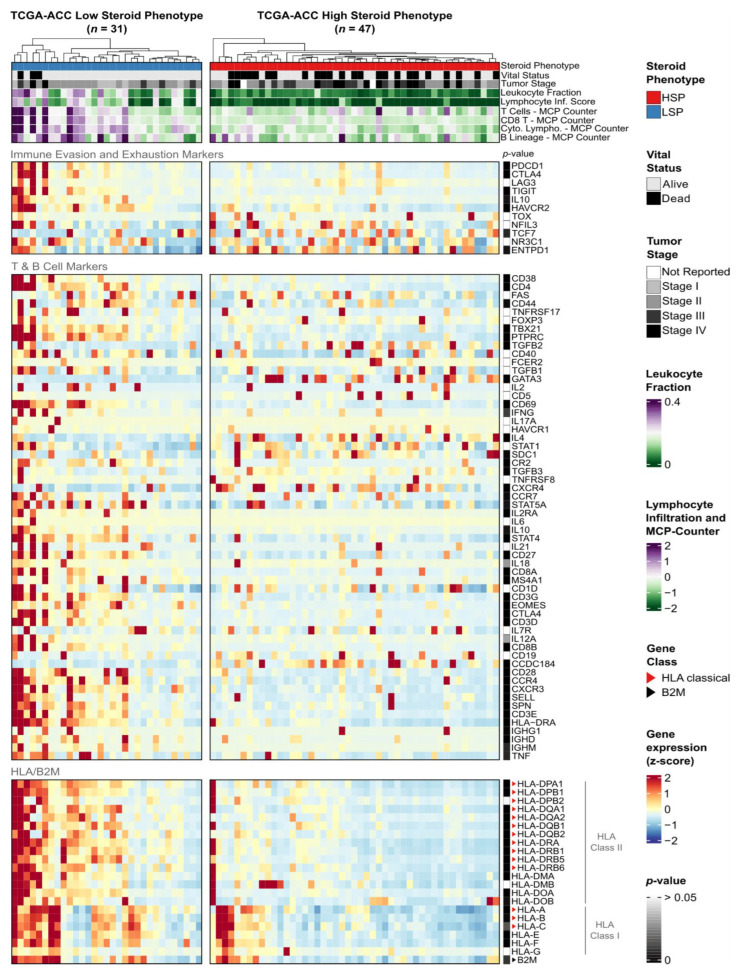
Functional characterization of the immune microenvironment in ACC subgroups. Heatmap displaying the expression of immune-related genes in TCGA-ACC tumors, stratified into LSP (*n* = 31) and HSP (*n* = 47). Columns represent individual samples, hierarchically clustered within each subgroup based on the expression of HLA/B2M genes. The figure is divided into four horizontal panels: immune evasion and exhaustion markers (**superior panel**), T and B cell activation markers (**middle panel**), and HLA/B2M expression (**bottom panel**). The annotation panel at the top displays clinical characteristics and immune scores. Gene expression (TPM + 1) was normalized by z-score and scaled from −2 (blue) to +2 (red). The sidebar indicates Benjamini–Hochberg adjusted *p*-values from the Mann–Whitney test between LSP vs. HSP for each gene (dark gray indicates a low *p*-value; white indicates *p* > 0.05).

To further functionally characterize the tumor microenvironment (TME), we analyzed the expression of a panel of genes defining T and B lymphocyte activation and exhaustion, integrating these data with the HLA expression profile (Figure 5). This analysis further supports the dichotomy between the steroid phenotypes as being representative of a “hot” (LSP) versus “cold” (HSP) TME.

In LSP tumors, we observed higher expression of multiple genes associated with an active antitumor immune response compared to HSP. These included lymphocyte activation markers, genes defining a cytotoxic and Th1 response, and chemokines responsible for lymphocyte recruitment. Furthermore, the high expression of immunoglobulin genes points to an active B-cell humoral response. Collectively, these data suggest that the immune infiltrate in LSP tumors is functionally active and trends towards an antitumor profile.

Despite this, LSP tumors also exhibited high expression of multiple inhibitory receptors, which are classical markers of T-cell exhaustion. Genes such as PDCD1 (PD-1), CTLA4, HAVCR2 (TIM-3), and TIGIT were all overexpressed in the LSP group. This dual profile, of high activation and high exhaustion, appears to be the classical signature of a TME that has recognized the tumor and mounted a chronic response, but one that is being actively restrained by immune checkpoint pathways.

In contrast, some immune activation and exhaustion signatures were absent in the HSP group. The generalized low expression of these markers suggests a “cold” and immune desert phenotype for these tumors.

**Figure 6:**
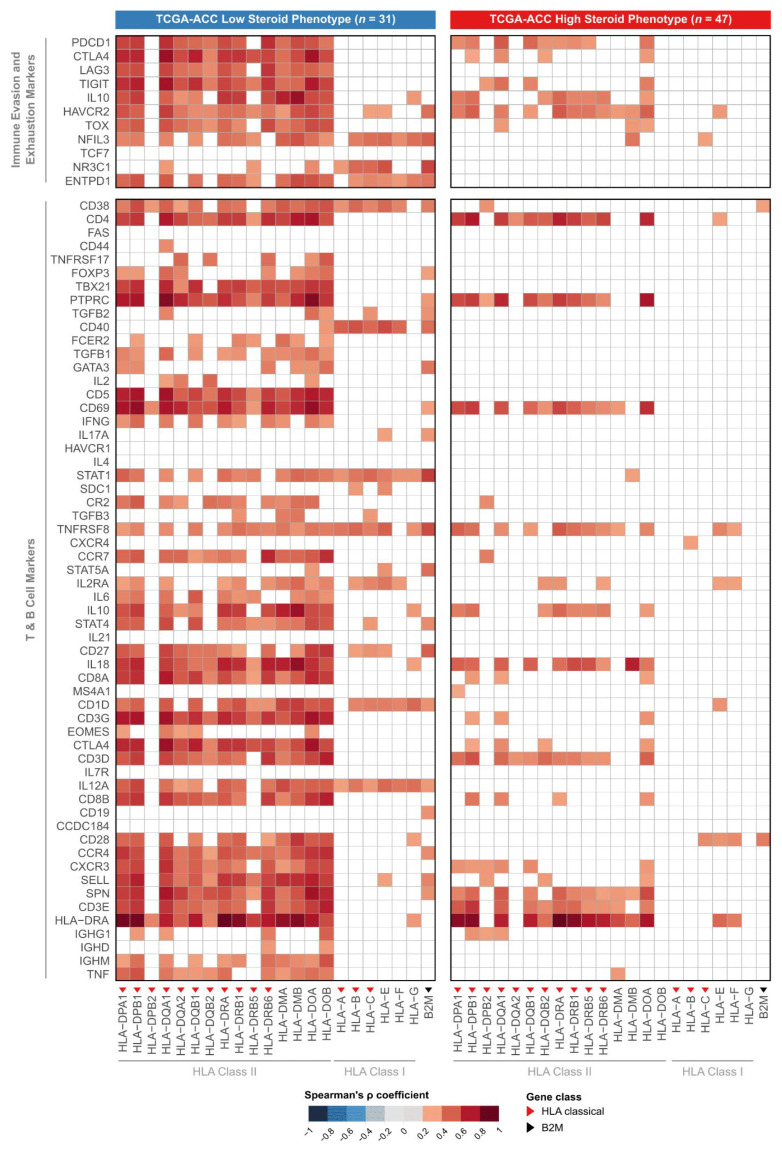
Functional profiling of immune-related genes with dual signature. Heatmaps display the Spearman correlation coefficients (ρ) between the expression of HLA/B2M genes (x-axis) and the expression of immune evasion/exhaustion and T/B cell marker genes (y-axis). The analysis was performed separately for the low steroid production (LSP, *n* = 31 (**left panel**)) and high steroid production (HSP, *n* = 47 (**right panel**)) subgroups. Color indicates the strength of the positive correlation, with darker red representing the strongest correlations. White cells indicate non-significant correlations (considering *p*-value adjustment by the Benjamini–Hochberg method). Red triangles indicate classic HLA Class I or II genes, and the black triangle indicates the B2M gene.

To statistically determine whether this immune activation and exhaustion signature is linked to the HLA gene profile, we correlated the expression of genes from the immune marker panel with the expression of HLA/B2M genes within each ACC subgroup (Figure 6).

An analysis of the LSP subgroup revealed a cluster of strong, positive correlations. The expression of almost all HLA Class I and II genes demonstrated a significant association with the expression of genes for T cell activation, cytotoxicity, Th1 response, and immune exhaustion markers. This result suggests that, in the LSP group, higher HLA gene expression correlates with higher expression of genes associated with T-cell response activation and exhaustion.

Fewer significant correlations were observed in the HSP subgroup, indicating that HLA expression may not be associated with the functional state of the immune microenvironment. Together, these findings provide evidence that the steroid phenotype appears to indicate the presence or absence of a functional immune response in ACC.

### 3.8. The Prognostic Value of HLA Expression Is Overshadowed by the Stronger Predictive Power of Tumor Stage and Steroid Phenotype

We investigated the clinical relevance of our findings by evaluating the association of the steroid phenotype, tumor stage, and HLA expression with overall survival (OS) in the TCGA-ACC cohort and assessing the consistency of these associations in the independent ENSAT-ACC cohort.

**Figure 7:**
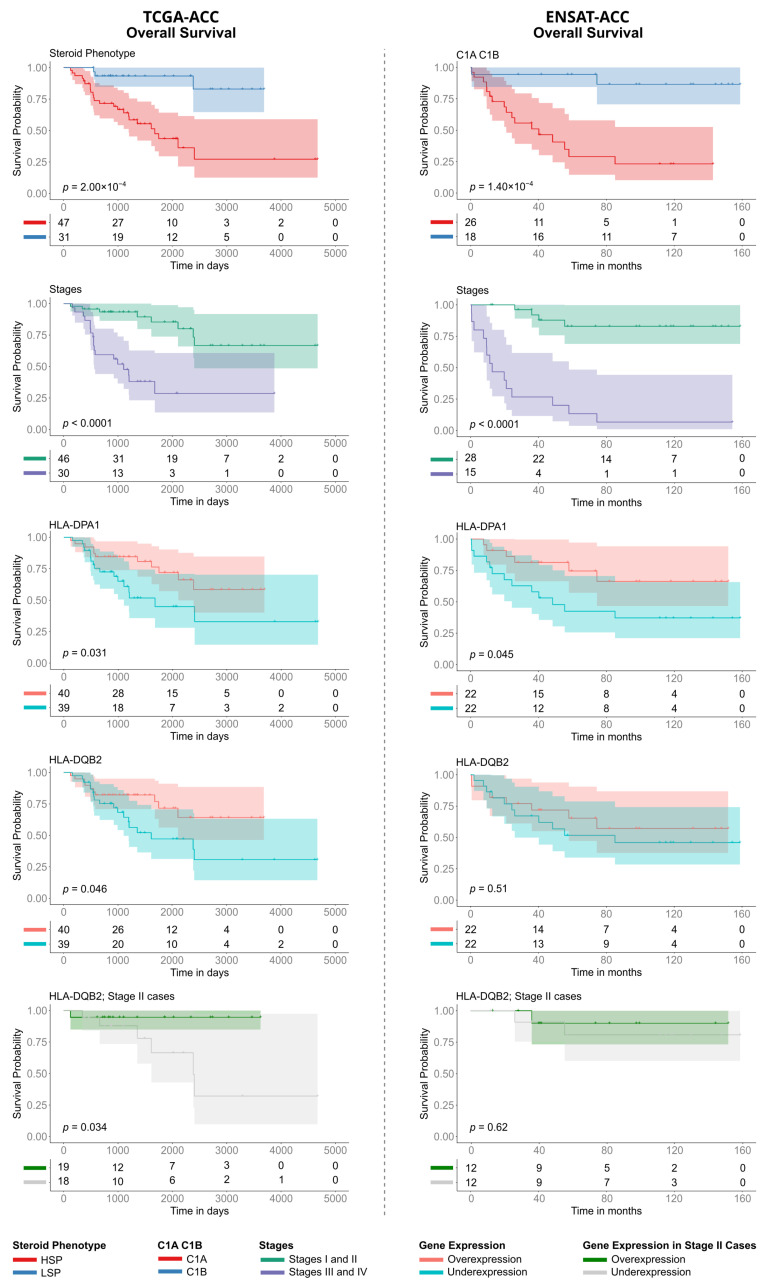
Kaplan–Meier analysis of Overall Survival (OS) in TCGA-ACC and ENSAT-ACC cohorts. The panels compare prognostic factors between the TCGA-ACC ((**left column**), time in days) and ENSAT-ACC ((**right column**), time in months) datasets. Survival curves are stratified by: (Row 1) Steroid phenotype (LSP versus HSP) for TCGA-ACC and Molecular Subtype (C1B versus C1A) for ENSAT-ACC; (Row 2) Tumor Stage (grouping Stages I/II versus Stages III/IV); (Rows 3 and 4) Expression levels of HLA-DPA1 and HLA-DQB2 (stratified into over- versus underexpression); and (Row 5) HLA-DQB2 expression specifically within the subset of Stage II patients. *p*-values were calculated using the log-rank test. The tables below each plot indicate the number of patients at risk at each time point.

Stratification by steroid phenotype (in TCGA) and C1A/C1B molecular subtype (in ENSAT) revealed significant survival differences (Figure 7, row 1). In the TCGA cohort, LSP patients had significantly longer survival compared to those with HSP (log-rank test, *p* = 2 × 10^−4^). This survival advantage was mirrored when stratifying the TCGA cohort by molecular subtype, where the C1B subgroup exhibited superior OS compared to C1A (Supplementary Figure S8, *p* < 0.0001). This pattern was validated in ENSAT-ACC cohort, where the C1B subgroup (LSP-like) exhibited superior OS compared to C1A (HSP-like) (*p* = 1.40 × 10^−4^), confirming the prognostic relevance of these overlapping phenotypes. Similarly, patients with early-stage tumors (I and II) had a better prognosis than those with advanced-stage disease (III and IV) in both cohorts (*p* < 0.001) (Figure 7, row 2).

Regarding individual gene expression in the TCGA-ACC cohort, only the Class II genes HLA-DQB2 and HLA-DPA1 were significantly associated with survival. High expression of HLA-DQB2 (*p* = 0.046) and HLA-DPA1 (*p* = 0.031) indicated a better prognosis (Figure 7, rows 3 and 4), though the associations were marginally significant. Analysis of the ENSAT cohort confirmed the prognostic trend for HLA-DPA1 (*p* = 0.045), while HLA-DQB2 did not reach significance (*p* = 0.51). To investigate whether the prognostic value of HLA expression was independent of tumor stage, we performed a stratified analysis. Notably, even within the subgroup of TCGA-ACC patients with Stage II tumors, which contains a balanced number of HSP (*n* = 18) and LSP cases (*n* = 19), high HLA-DQB2 expression continued showing a significant survival advantage (*p* = 0.034) (Figure 7, row 5).

To consolidate these findings, we used the HLA pathway signature score. Patients with a high HLA pathway signature score showed significantly better overall survival than those with a low score (*p* = 0.035; Supplementary Figure S9A). However, a more in-depth additive analysis revealed that prognosis was better explained by the steroid effect (Supplementary Figure S9B).

Therefore, to determine the independent prognostic factors, we constructed a multivariate Cox proportional hazards model using the TCGA-ACC data. In this analysis, which simultaneously adjusted for the HLA pathway signature score, tumor stage, and steroid phenotype, only advanced tumor stage (HR = 12.80, *p* = 0.019) and the LSP phenotype (HR = 0.14, *p* = 0.01) remained as independent and significant predictors of survival (Supplementary Figure S9C). The HLA pathway signature score did not retain independent statistical significance (*p* = 0.215), suggesting that its prognostic value is largely contained within the superior predictive power of the steroid phenotype. Consistently, when testing the expression of each individual HLA gene in multivariate Cox models (adjusted for steroid phenotype and tumor stage), no HLA gene retained prognostic significance after correction for multiple testing (Table 2).

## 4. Discussion

In this study, we show that stratifying ACC by steroid production phenotype uncovers two fundamentally distinct tumor immune landscapes [8,30]. HSP is characterized by suppressed HLA expression and an immunologically “cold” TME, while LSP displays enhanced HLA expression and a “hot,” inflamed TME. The robustness of this steroid-associated HLA signature is underscored by our in silico validation in the independent ENSAT-ACC cohort. Despite the smaller sample size (*n* = 44) and the technical differences between microarray (ENSAT) and RNA-seq (TCGA) profiling, the core pattern of HLA downregulation in the high-risk C1A (the HSP-like) subtype was largely preserved. Furthermore, this inflammatory state was supported by the validation cohort, where the low-risk C1B (the LSP-like) subtype also exhibited significantly higher infiltration scores for cytotoxic lymphocytes. These findings indicate that the steroid phenotype is a central determinant of immune visibility in ACC, modulating both the magnitude of HLA expression and its capacity to drive T-cell recruitment and activation, with direct implications for prognosis and therapeutic stratification [12,17,30,47,48,49].

Transcriptomic stratification was prioritized over clinical hormone levels due to reported discrepancies between intratumoral steroid content and peripheral circulation [24]. This molecular “fingerprint” accounts for the fact that tissue-level concentrations are often higher than blood levels, providing a more direct determinant of the tumor microenvironment while minimizing inconsistencies inherent in systemic clinical assays.

Our pan-cancer comparison refines previous observations by Schaafsma et al. (2021) [20], who described ACC as a tumor with generally low HLA expression. By stratifying the cohort, we demonstrate that this signal is driven specifically by the HSP subgroup, which ranked as the lowest of 34 tumor types. In contrast, the LSP subgroup repositioned to an intermediate level, clustering with more immunogenic solid tumors. This observation aligns with the TCGA pan-cancer immune subtype classification [25]: HSP tumors were enriched for the lymphocyte-depleted (C4) subtype, while LSP tumors were enriched for the inflammatory (C3) subtype.

The suppression of HLA pathway genes in HSP tumors is consistent with the known immunosuppressive actions of glucocorticoids. Cortisol has well-documented effects on antigen presentation, including downregulation of HLA expression [16,50,51]. In pancreatic cancer, glucocorticoid receptor signaling has been shown to regulate transcription of HLA class I genes [52]. Our data therefore support a model in which endogenous cortisol excess in HSP directly drives immune evasion by attenuating antigen presentation (Supplementary Figure S3).

HLA expression is functionally coupled to immune infiltration only in LSP. The consequences of this steroid-driven regulation were evident in our immune analyses. In LSP tumors, higher HLA expression coincided with greater lymphocyte infiltration and correlated strongly with the presence of T cells and signatures of a Th1/cytotoxic response. In HSP tumors, however, HLA expression did not correlate with immune infiltration, reflecting a “silent” antigen presentation pathway within a cortisol-rich microenvironment. This functional uncoupling explains the immune desert phenotype of HSP and further highlights the LSP subgroup as immunologically distinct.

A hallmark of the LSP phenotype was the coexistence of immune activation and exhaustion. LSP tumors showed high expression of genes associated with cytotoxicity, Th1 polarization, and B-cell activity, alongside overexpression of inhibitory checkpoints such as PD-1 (PDCD1), CTLA-4, TIM-3 (HAVCR2), and TIGIT. This dual pattern represents the classic “inflamed but exhausted” TME seen in immunogenic cancers [53,54]. In contrast, HSP tumors lacked both activation and exhaustion signatures, consistent with primary resistance to immunotherapy [55,56,57]. These results suggest that LSP patients may benefit from immune checkpoint inhibitors (ICIs), while HSP patients may require approaches targeting steroidogenesis or immune exclusion mechanisms.

Our survival analyses confirm the steroid phenotype as a major independent prognostic factor, with LSP conferring significantly longer survival even after adjustment for tumor stage. This prognostic dichotomy was corroborated in the ENSAT cohort, where the C1B (LSP-like) subtype exhibited significantly superior survival compared to C1A (HSP-like). Although high expression of some HLA class II genes (e.g., HLA-DPA1, HLA-DQB2) was associated with improved outcomes, these effects did not remain independently significant in multivariate analyses (Supplementary Figure S9C). The weaker prognostic value of HLA expression may reflect both its mechanistic dependence on the steroid phenotype and the limited sample size of the TCGA cohort, which constrains statistical power. Importantly, this suggests that HLA expression acts as a mediator of steroid phenotype effects on the TME, rather than as an independent prognostic marker or standalone biomarker [12,58,59].

The consistent downregulation of HLA class II genes (HLA-DPA1, HLA-DPB1, HLA-DRA) is noteworthy, as these same genes have been linked to worse prognosis in pediatric ACC [44,45,46]. Our data extend these observations to adults, showing similar dysregulation in HSP tumors and a worse survival trend for low HLA-DPA1 expression. The differential expression of these three specific genes was successfully validated in the ENSAT-ACC cohort, where they were also found to be significantly upregulated in the LSP-like C1B subtype compared to the HSP-like C1A. This convergence is striking given the differing etiologies of pediatric (often TP53-related) and adult (mostly sporadic) ACC [60], suggesting that HLA class II suppression represents a common immune escape mechanism across disease subtypes.

This study has limitations. It is retrospective and based on bulk transcriptomic data, which limits cell-type resolution and may obscure heterogeneity within the TME. In addition, while the ENSAT cohort provided crucial validation, the smaller sample size and the use of different molecular platforms (RNA-Seq versus microarray) limit statistical power and introduce technical variability that must be considered when interpreting the magnitude of gene expression differences. The downregulation of HLA class II genes in the HSP cluster should be interpreted alongside its higher tumor purity and significantly lower stromal fraction. These results suggest that the observed transcriptomic differences are likely driven by the lower density of infiltrating immune cells in this subgroup, rather than an isolated suppression of these genes within the malignant cells themselves. It should also be noted that the HSP/LSP classification, based on a transcriptional signature of steroidogenic genes [8], did not show complete concordance with the excess cortisol groups, as five LSP-classified tumors presented with cortisol excess. This discordance likely reflects the dissociation between systemic secretion and the local tumor environment, potentially due to variable steroid export kinetics or the secretion of intermediate precursors not captured by standard clinical cortisol assays [24]. In these instances, transcriptomic signatures may provide a more direct indicator of the local immunosuppressive state than peripheral measurements.

Although these analyses provide a comprehensive view of the ACC immune landscape, the proposed mechanisms are inferred from correlations and prior literature. Consequently, the absence of protein-level validation (e.g., immunohistochemistry) and functional assays remains a primary limitation. Future studies integrating single-cell RNA sequencing (scRNA-seq), spatial profiling, and immunohistochemistry for HLA proteins and CD8^+^ T cells will be critical to resolve per-cell expression levels and confirm the specific biological drivers of these associations. Finally, prospective clinical studies should test whether steroid phenotype stratification can refine patient selection for immunotherapy in ACC. While the C1A/C1B classification is a powerful prognostic tool, the steroidogenic profile can provide a link to immune evasion. Identifying “cold” tumors driven by steroid-mediated mechanisms can allow for targeted strategies, such as combining steroidogenesis inhibitors with immunotherapy, that are not explicitly indicated by broader subtyping. From a translation perspective, while blood measurements remain most accessible, integrating them with direct intratumoral assessments would allow clinicians to account for the actual local metabolic state, potentially refining patient selection for these emerging combination therapies.

Altogether, our findings position the steroid phenotype as a central biomarker in ACC, distinguishing tumors into immunologically “hot” (LSP) and “cold” (HSP) categories [8,12,61]. This endocrine-driven mechanism of immune escape, mediated in part through regulation of HLA gene expression, explains both the variability in tumor immunogenicity and the survival differences between phenotypes. Clinically, these insights provide a strong rationale for incorporating steroid phenotype into prognostic models and therapeutic decision-making and for developing phenotype-tailored immunotherapy strategies in this rare and aggressive malignancy.

## 5. Conclusions

In summary, these findings suggest that the steroid phenotype contributes to the immunological distinction between ‘hot’ (LSP) and ‘cold’ (HSP) categories in ACC. This potential endocrine-linked mechanism of immune escape, possibly mediated through the regulation of HLA expression, may help clarify the observed variability in tumor immunogenicity and patient survival.

In conclusion, our study characterized the HLA expression profiles of ACC stratified by steroid phenotype and evaluated their potential associations with the immune microenvironment and patient outcomes. By exploring the relationship between the steroidogenic metabolic profile and immune escape, we identify a specific subset of patients who may potentially benefit from tailored immunotherapeutic strategies. These insights suggest that integrating steroid profiling into clinical decision-making could help refine prognostic accuracy and therapeutic selection in this aggressive malignancy.

## Figures and Tables

**Table 1:** Clinicopathological and immunological characteristics of the TCGA-ACC cohort stratified by steroid phenotype. The table summarizes the characteristics of the 78 ACC cases for which steroid phenotype information was available. Values for categorical variables are presented as patient count (*n*), percentage of the subgroup total (%), and number of deaths, in the format *n* (%) ^†^deaths. Age is presented as mean ± standard deviation. Bold font denotes primary clinical categories and demographic headings to distinguish them from their respective subcategories or specific levels. Abbreviations: HSP, High Steroid Production; LSP, Low Steroid Production; SD, Standard Deviation.

	HSP	LSP
**Total**	47 (100%) ^†^24	31 (100%) ^†^3
**Male**	16 (34%) ^†^9	15 (48%) ^†^2
**Female**	31 (66%) ^†^15	16 (52%) ^†^1
**Mean Age ± SD (Total)**	46.5 ± 17	48.5 ± 14.3
**Mean Age ± SD (Male)**	46 ± 14.6	52.9 ± 11
**Mean Age ± SD (Female)**	46.8 ± 18.3	44.3 ± 16.1
**Tumor Stage**		
Stage I	3 (6%) ^†^1	6 (19%) ^†^0
Stage II	18 (38%) ^†^6	19 (61%) ^†^1
Stage III	12 (26%) ^†^6	4 (13%) ^†^1
Stage IV	12 (26%) ^†^10	2 (6%) ^†^1
**Hormone Excess**		
Cortisol	27 (57%) ^†^14	5 (16%) ^†^1
Mineralocorticoids	3 (6%) ^†^1	1 (3%) ^†^0
Sexual steroids	20 (43%) ^†^14	8 (26%) ^†^1
**C1A/C1B**		
C1A	40 (85%) ^†^22	3 (10%) ^†^2
C1B	7 (15%) ^†^2	28 (90%) ^†^1
**Immune Subtype**		
C1—Wound Healing	1 (2%) ^†^1	0 (0%) ^†^0
C2—IFN-y Dominant	0 (0%) ^†^0	1 (3%) ^†^1
C3—Inflammatory	7 (15%) ^†^3	16 (52%) ^†^0
C4—Lymphocyte Depleted	37 (79%) ^†^19	12 (39%) ^†^1
C5—Immunologically Quiet	2 (4%) ^†^1	1 (3%) ^†^0
C6—TGF-B Dominant	0 (0%) ^†^0	1 (3%) ^†^1

**Table 2:** Multivariate survival analysis for individual HLA genes. The table shows the results of multivariate Cox proportional hazards models for each HLA/B2M gene (HLA gene expression adjusted for the covariates steroid phenotype and tumor stage). For each gene, the Hazard Ratio (HR), 95% confidence intervals (CI; lower and upper limits) for the HR, the raw *p*-value, and the *p*-value adjusted by the Benjamini–Hochberg method are presented.

Gene	HR	Lower CI	Upper CI	*p*-Value	Adjusted *p*-Value
HLA-DPA1	1.24	0.92	1.68	0.16	0.54
HLA-DPB1	1.24	0.89	1.73	0.20	0.54
HLA-DPB2	1.01	0.55	1.86	0.97	0.97
HLA-DQA1	1.22	0.84	1.77	0.30	0.59
HLA-DQA2	0.94	0.70	1.26	0.68	0.85
HLA-DQB1	1.17	0.87	1.57	0.31	0.59
HLA-DQB2	1.16	0.76	1.76	0.49	0.74
HLA-DRA	1.20	0.89	1.61	0.23	0.54
HLA-DRB1	1.28	0.96	1.70	0.09	0.54
HLA-DRB5	1.09	0.87	1.38	0.45	0.73
HLA-DRB6	1.21	0.91	1.62	0.19	0.54
HLA-DMA	1.31	0.89	1.95	0.17	0.54
HLA-DMB	1.48	1.08	2.03	0.01	0.30
HLA-DOA	1.17	0.80	1.72	0.41	0.71
HLA-DOB	2.68	1.05	6.84	0.04	0.41
HLA-A	1.07	0.71	1.59	0.75	0.88
HLA-B	1.03	0.75	1.41	0.86	0.90
HLA-C	1.10	0.78	1.54	0.59	0.83
HLA-E	1.06	0.67	1.67	0.80	0.88
HLA-F	1.08	0.74	1.58	0.69	0.85
HLA-G	0.78	0.53	1.15	0.21	0.54

## Data Availability

All data and software used in this study are publicly available in the sources described in Section 2. All scripts and R data to generate results for this study are available on the GitHub repository https://github.com/igorginer/Sup_Material_Giner2025 (accessed on 9 January 2026).

## Supplementary Materials

The following supporting information can be downloaded at https://www.mdpi.com/article/10.3390/cancers18020229/s1, Supplementary Figure S1: Pan-cancer comparison of the expression of HLA genes associated with pediatric adrenocortical tumors; Supplementary Figure S2: Unsupervised hierarchical clustering of HLA/B2M gene expression in the TCGA-ACC cohort; Supplementary Figure S3: Differential Expression of HLA/B2M Genes Between ACC Steroid Phenotypes in the TCGA-ACC cohort; Supplementary Figure S4: Differential Expression of HLA/B2M Genes Between ACC C1A/C1B molecular subtypes in the ENSAT-ACC cohort; Supplementary Figure S5: Differential Expression of HLA/B2M Genes Between ACC C1A/C1B molecular subtypes in the TCGA-ACC cohort; Supplementary Figure S6: Differentially expressed genes between C1A and C1B in both TCGA-ACC and ENSAT-ACC cohorts; Supplementary Figure S7: Quantitative analysis of clinical and microenvironmental features in TCGA-ACC and assessment of immune infiltration in ENSAT-ACC; Supplementary Figure S8: Evaluation of the prognostic impact of C1A/C1B molecular subtypes on survival in TCGA-ACC patients; Supplementary Figure S9: Multivariate survival analysis in TCGA-ACC; Supplementary Table S1: Sample counts for each cancer type in the TCGA Pan-Cancer (PANCAN) dataset; Supplementary Table S2: Clinicopathological characteristics of the ENSAT-ACC cohort stratified by C1A/C1B molecular subtypes.
