# Supplementary material for: Steroid Phenotype Stratification Reveals Distinct HLA Expression Signatures in Adrenocortical Carcinoma
Source: Cancers (Basel). 2026 Jan 12;18(2):229. doi: 10.3390/cancers18020229 (PMC12839242; doi:10.3390/cancers18020229)
Supplement: Supplementary file 1 [file cancers-18-00229-s001.zip › cancers-4066331-supplementary.pdf]

MDPI

cancers

Supplementary Materials

Article

Steroid Phenotype Stratification Reveals Distinct HLA Expression Signatures in Adrenocortical Carcinoma

Igor S. Giner [1,2,3], Jean S. S. Resende [1,2,3], João C. D. Muzzi [1,2,3], José A. M. Barbuto [4,5], Enzo Lalli [6], Mauro A. A. Castro [3,*] and Bonald C. Figueiredo [1,2,*]

1 Instituto de Pesquisa Pelé Pequeno Príncipe, Oncology Division, Curitiba, PR 80250-060, Brazil; igor.giner@aluno.fpp.edu.br, jean.resende@professor.fpp.edu.br, jmuzzi@quantumds.tech
2 Faculdades Pequeno Príncipe, Av. Iguaçu, 333 - Rebouças, Curitiba - PR, 80230-020, Brazil
3 Bioinformatics and Systems Biology Laboratory, Federal University of Paraná, Curitiba, PR 81520-260, Brazil
4 Department of Immunology, Institute of Biomedical Sciences, University of São Paulo, São Paulo, SP 05508-000, Brazil; jbarbuto@icb.usp.br
5 Laboratory of Medical Investigation in Pathogenesis and Targeted Therapy in Onco-Immuno-Hematology (LIM-31), Department of Hematology, Hospital das Clínicas HCFMUSP, Faculty of Medicine, University of São Paulo, São Paulo, SP 05403-000, Brazil
6 Institut de Pharmacologie Moléculaire et Cellulaire CNRS UMR7275, Inserm U1323, Université Côte d'Azur, 06560 Valbonne, France; lalli@ipmc.cnrs.fr
* Correspondence: bonaldf@yahoo.com.br; bonald.figueiredo@professor.fpp.edu.br (B.C.F); mauro.a.castro@gmail.com (M.A.A.C.)

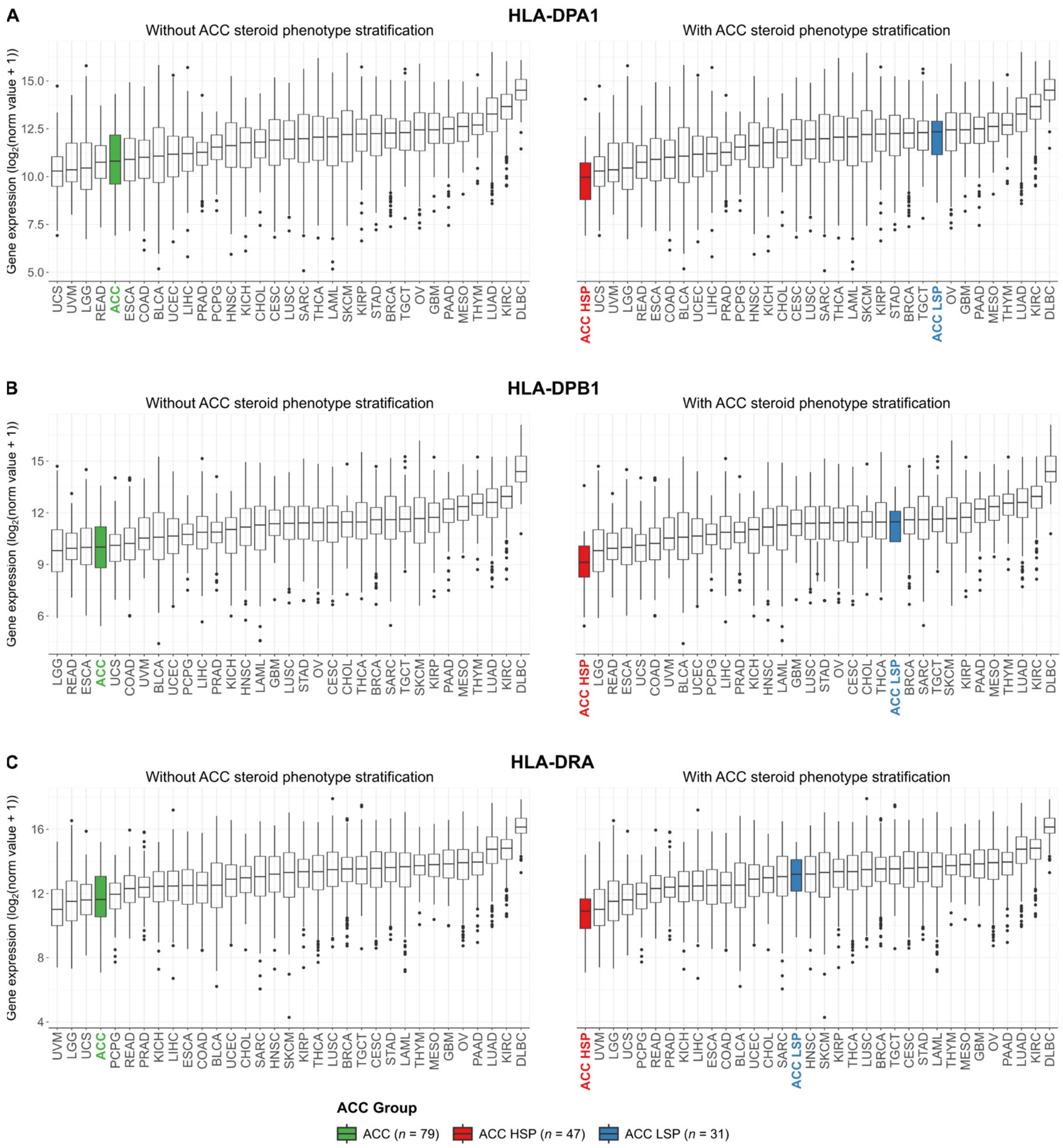

Figure S1. Pan-cancer comparison of the expression of HLA genes associated with pediatric adrenocortical tumors. Boxplots show the expression in $\log_2$(normalized values + 1) across 33 TCGA cancer types for (A) HLA-DPA1, (B) HLA-DPB1, and (C) HLA-DRA. The cancer types are ordered by ascending median expression. After stratification by steroid phenotype, ACC samples are highlighted by color: green (unstratified ACC, *n* = 79), red (ACC HSP, *n* = 47), and blue (ACC LSP, *n* = 31). Stratification was based on Zheng et al. (2016) [8]. The boxplots indicate the median (line), the interquartile range (box), and the whiskers (1.5x IQR); points are outliers.

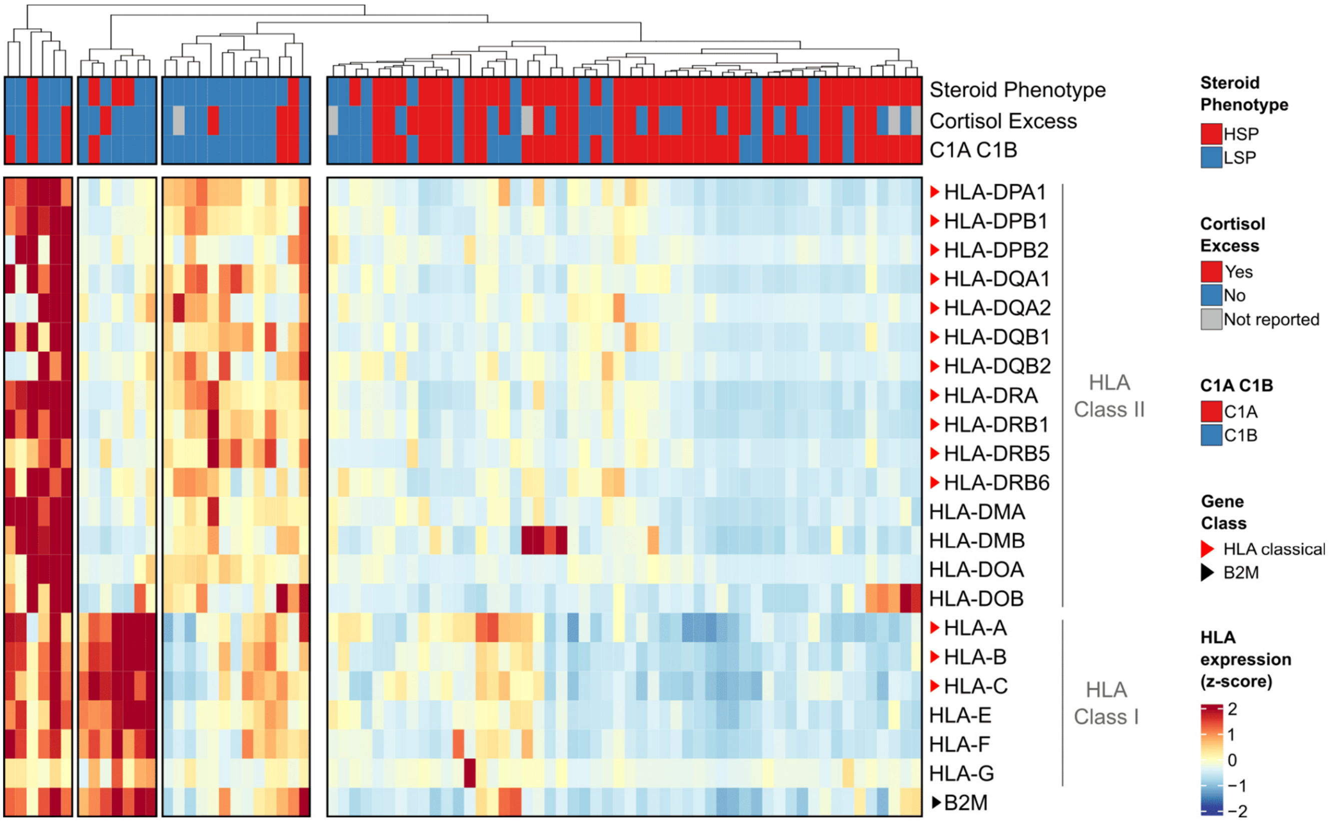

Figure S2. Unsupervised hierarchical clustering of HLA/B2M gene expression in the TCGA-ACC cohort. Heatmap displaying the transcriptomic grouping of 78 TCGA-ACC samples. This unsupervised analysis was performed using Euclidean distance and the complete linkage method. The heatmap was generated using the ComplexHeatmap R package [31]. Samples (columns) are grouped into four main clusters. The annotation panels at the top show the distribution of the previously defined steroid phenotype (LSP and HSP), cortisol excess status, and molecular subtype (C1A and C1B). Gene expression values are represented as z-scores, ranging from -2 (blue, lower expression) to +2 (red, higher expression). Red triangles denote classical Class I or II HLA genes, and the black triangle denotes the B2M gene.

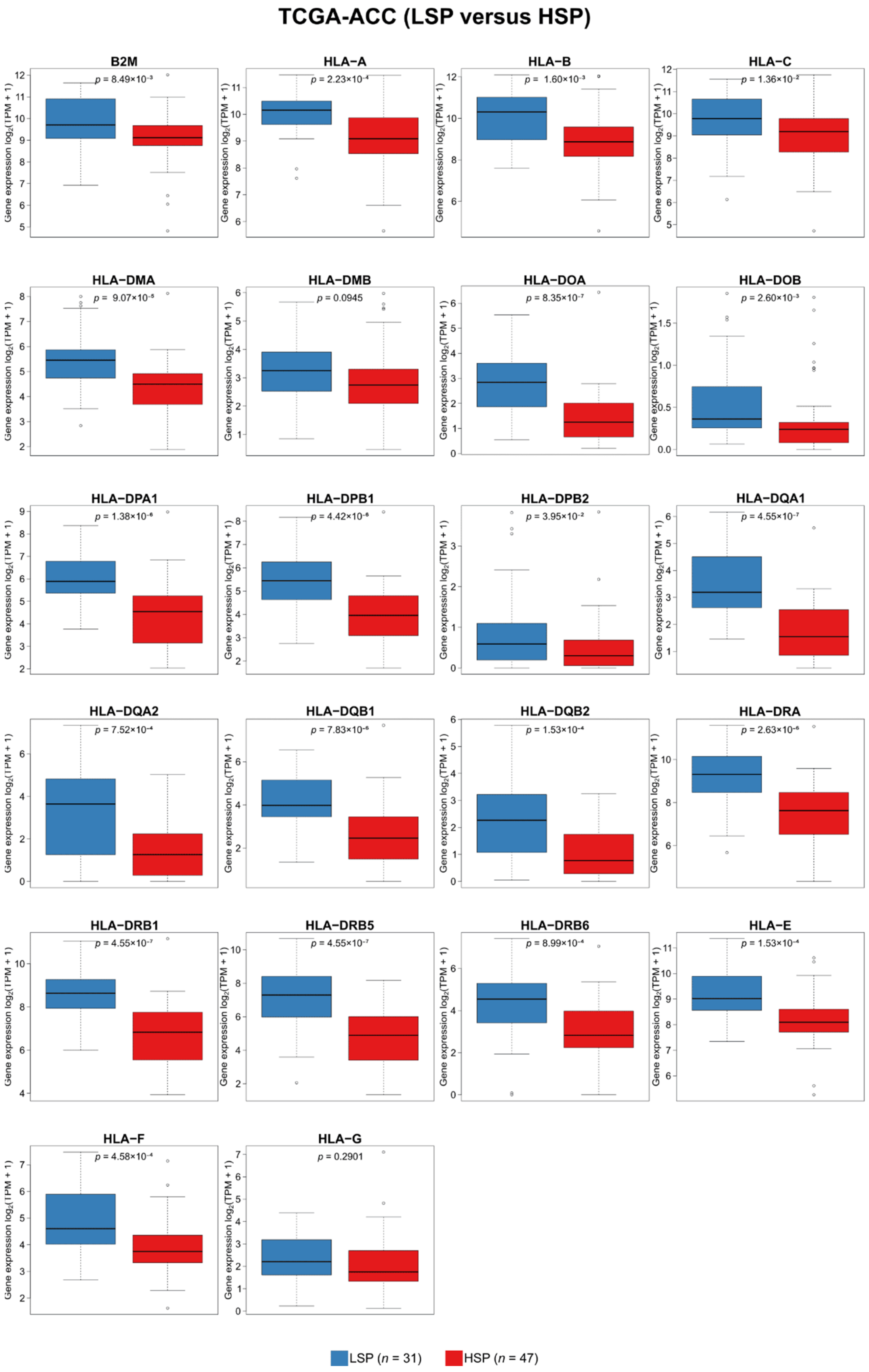

Figure S3. Differential Expression of HLA/B2M Genes Between ACC Steroid Phenotypes in the TCGA-ACC cohort. Boxplots compare the expression levels [$\log_2$(TPM + 1)] of 22 individual genes from the antigen presentation pathway between the low steroid production (LSP, n = 31, in blue) and high steroid production (HSP, n = 47, in red) subgroups of the TCGA-ACC cohort. Each panel represents one gene. Boxes represent the interquartile range (IQR), the central line indicates the median, and whiskers extend up to 1.5× the IQR. Differences between subgroups for each gene were

statistically evaluated by the Mann-Whitney U test; significant adjusted *p*-values ($p < 0.05$) are indicated above the respective plots.

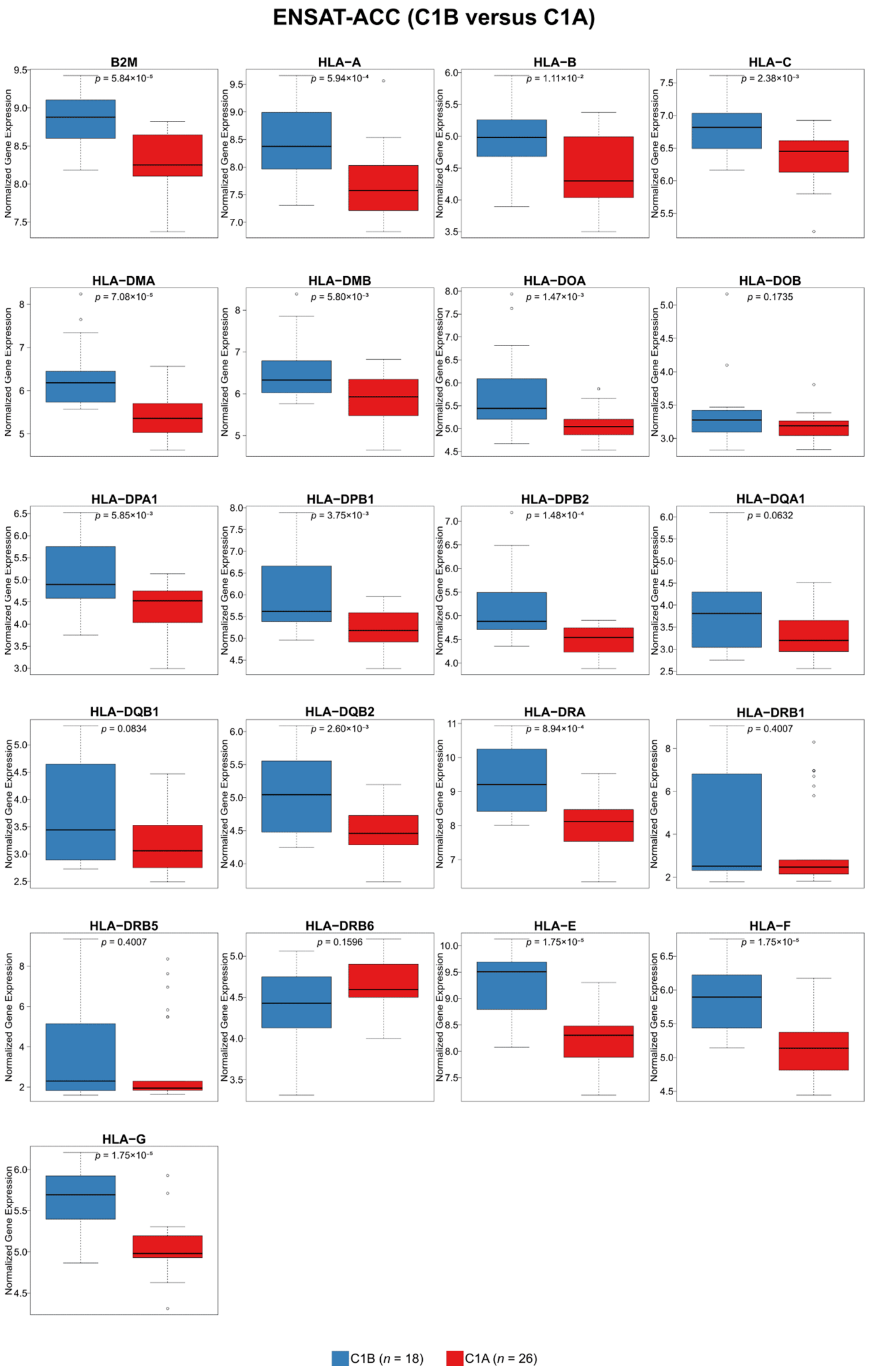

Figure S4. Differential Expression of HLA/B2M Genes Between ACC C1A/C1B molecular subtypes in the ENSAT-ACC cohort. Boxplots compare the expression levels (RMA normalized) of 21 individual genes from the antigen presentation pathway between the C1B ($n = 18$, in blue) and C1A ($n = 26$, in red) subgroups of the ENSAT-ACC cohort. Each panel represents one gene. Boxes represent

the interquartile range (IQR), the central line indicates the median, and whiskers extend up to 1.5× the IQR. Differences between subgroups for each gene were statistically evaluated by the Mann-Whitney U test; significant adjusted *p*-values ($p < 0.05$) are indicated above the respective plots.

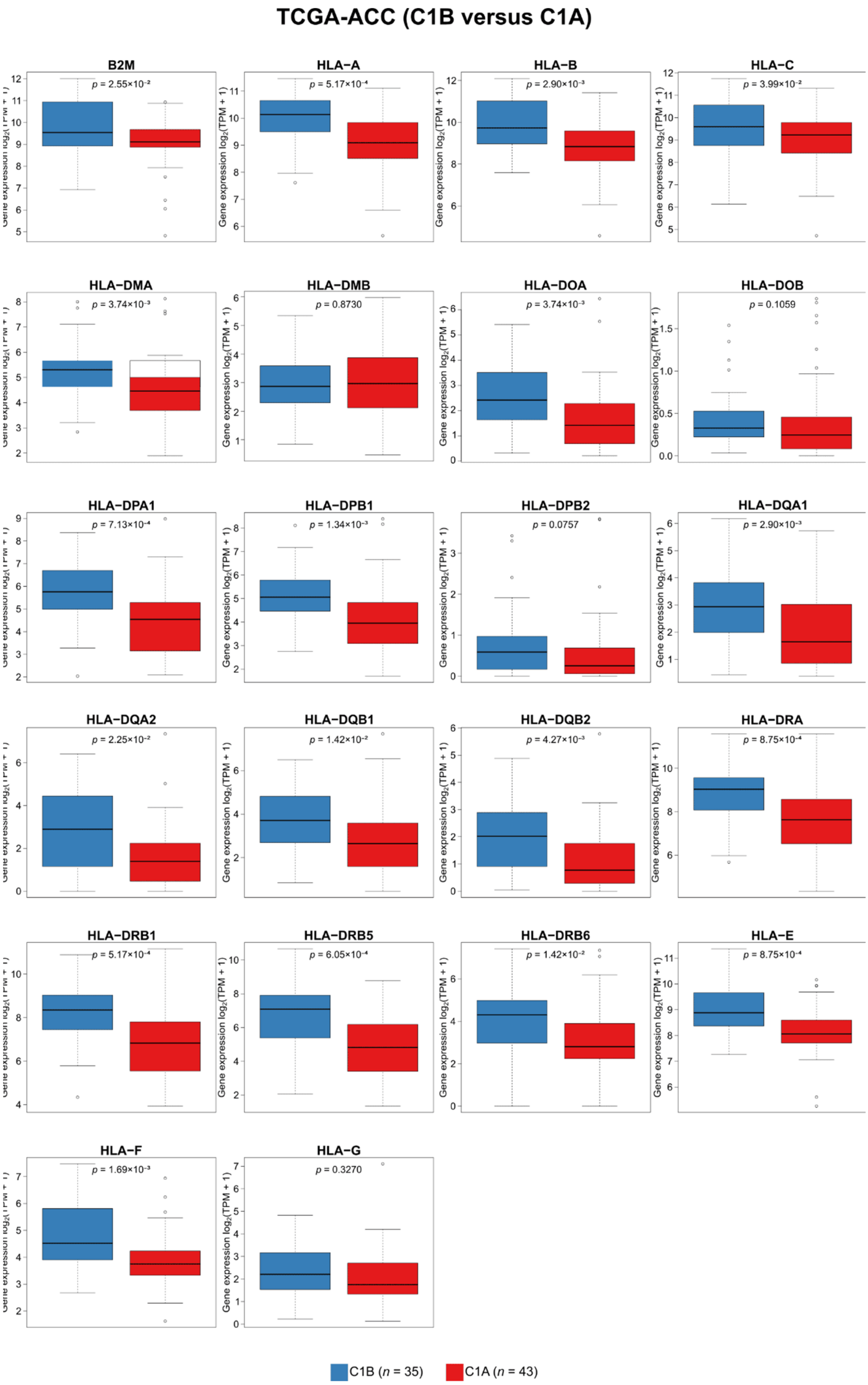

Figure S5. Differential Expression of HLA/B2M Genes Between ACC C1A/C1B molecular subtypes in the TCGA-ACC cohort. Boxplots compare the expression levels [$\log_2$(TPM + 1)] of 22 individual genes from the antigen presentation pathway between the C1B (n = 35, in blue) and C1A (n = 43, in

red) subgroups of the TCGA-ACC cohort. Each panel represents one gene. Boxes represent the interquartile range (IQR), the central line indicates the median, and whiskers extend up to 1.5× the IQR. Differences between subgroups for each gene were statistically evaluated by the Mann-Whitney U test; significant adjusted *p*-values ($p < 0.05$) are indicated above the respective plots.

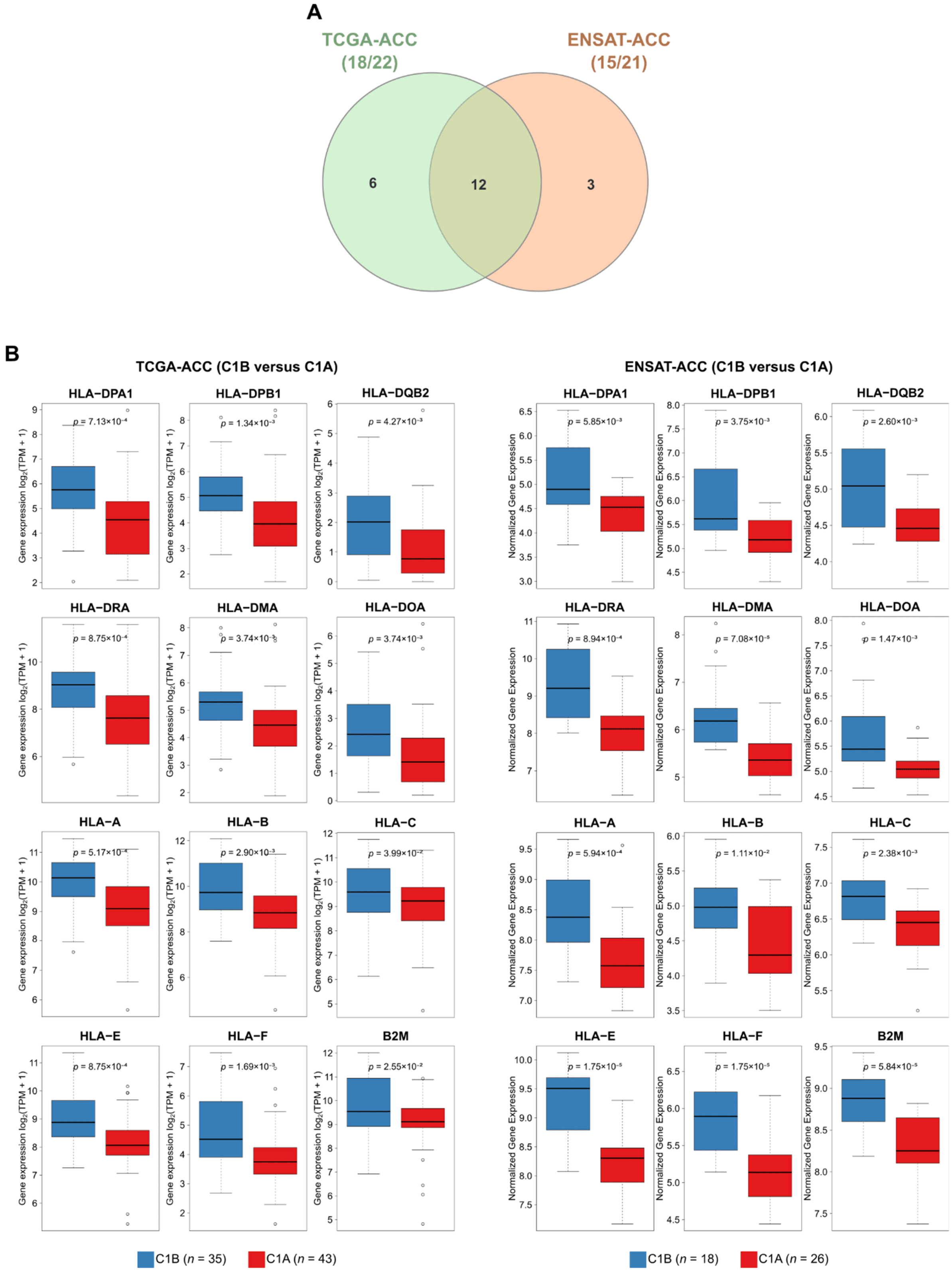

Figure S6. Differentially expressed genes between C1A and C1B in both TCGA-ACC and ENSAT-ACC cohorts. (**A**) Venn diagram illustrating the overlap of differentially expressed genes (DEGs)

identified between the C1A and C1B molecular subtypes both in the TCGA-ACC cohort (green circle, n = 18 DEGs out of 22 genes in total) and the ENSAT-ACC cohort (orange circle, n = 15 DEGs out of 21 genes in total). The intersection highlights 12 common DEGs. (**B**) Boxplots showing the expression distribution for these 12 common DEGs across C1B (blue) and C1A (red) subtypes in both the TCGA-ACC cohort (left panel, expression in [$\log_2$(TPM+1]) and the ENSAT-ACC cohort (right panel, expression in RMA normalized values). Benjamini-Hochberg adjusted p-values from the Mann-Whitney U tests are displayed above each boxplot.

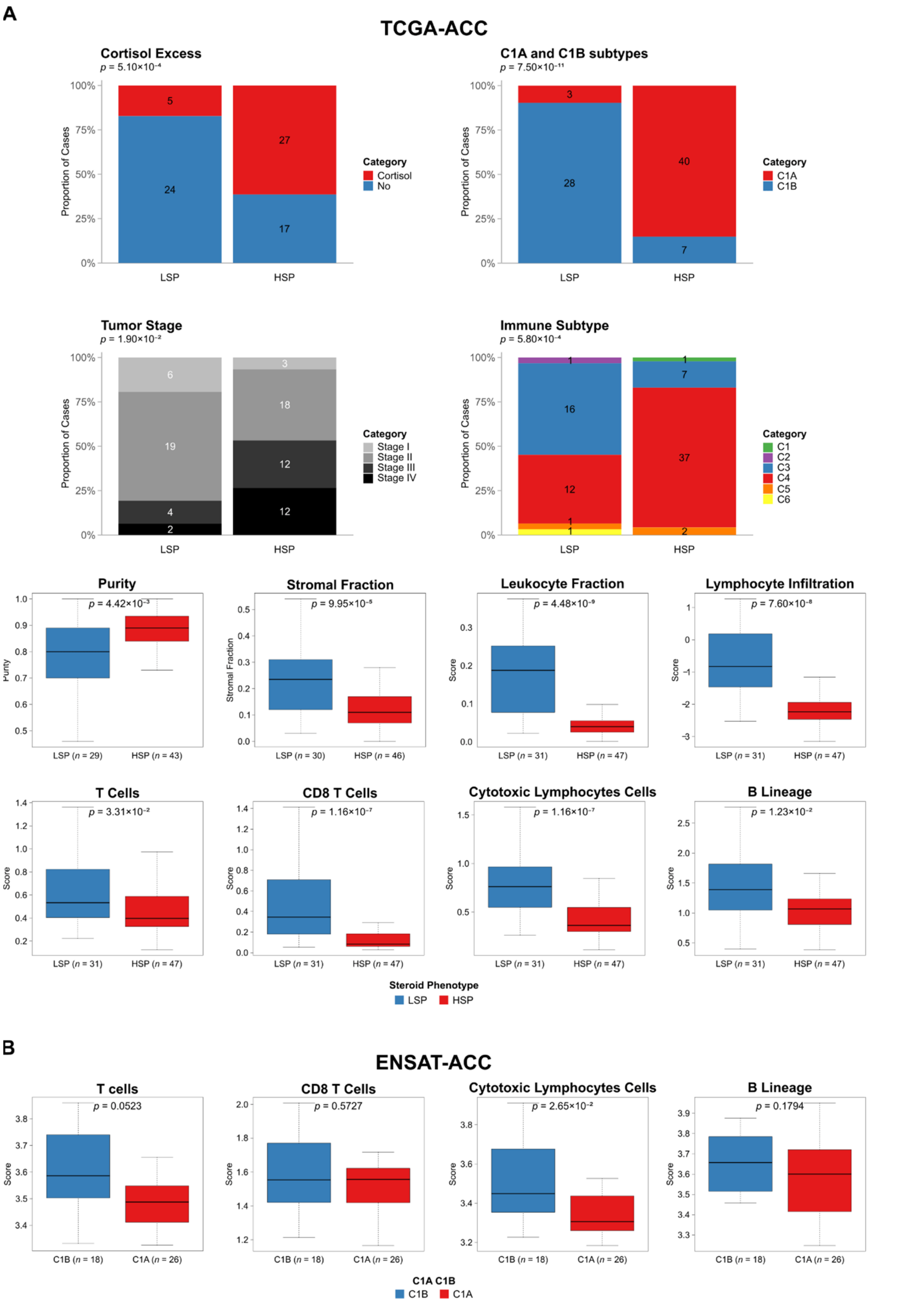

Figure S7. Quantitative analysis of clinical and microenvironmental features in TCGA-ACC and assessment of immune infiltration in ENSAT-ACC. (**A**) Statistical comparisons of key variables be-

tween LSP and HSP in the TCGA-ACC cohort. The stacked bar plots in the top row show the proportional distribution of cortisol excess, C1A/C1B molecular subtypes, tumor stage, and immune subtypes, with the significance of differences evaluated by Fisher's Exact Test and adjusted by the Benjamini-Hochberg method. The boxplots in the subsequent rows compare the distributions of tumor purity, stromal fraction, leukocyte fraction, and multiple lymphocyte infiltration scores (derived from MCP-counter), with significance evaluated by the Mann-Whitney U test and adjusted by the Benjamini-Hochberg method. (**B**) Boxplots comparing lymphocyte infiltration scores (derived from MCP-counter) between the C1A and C1B molecular subtypes in the ENSAT-ACC cohort. Significance was evaluated by the Mann-Whitney U test and adjusted by the Benjamini-Hochberg method). In each boxplot, boxes represent the interquartile range (IQR), the central line indicates the median, and whiskers extend up to 1.5× the IQR.

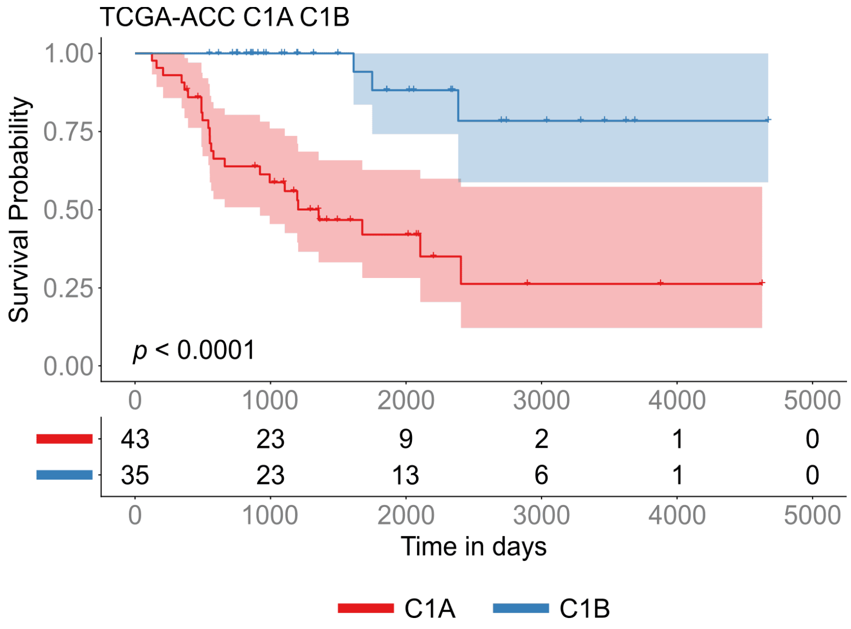

Figure S8. Evaluation of the prognostic impact of C1A/C1B molecular subtypes on survival in TCGA-ACC patients. Kaplan-Meier curve for overall survival in the TCGA-ACC cohort. Patients were stratified by ACC C1A/C1B molecular subtypes. The *p*-value indicated was calculated using the log-rank test. The table below the curve shows the number of patients at risk at each time interval.

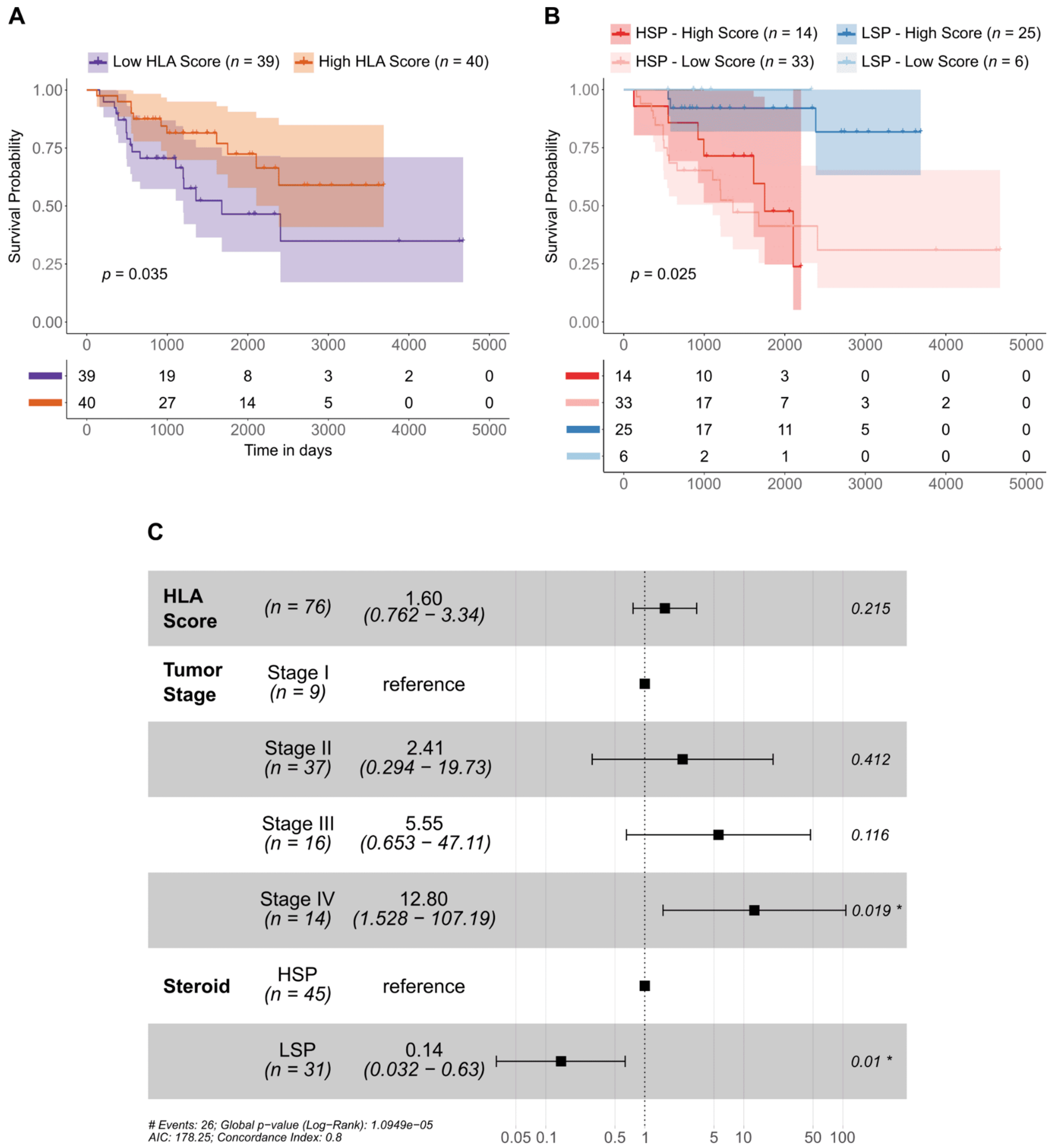

Figure S9. Multivariate Survival Analysis in TCGA-ACC. (**A**) Kaplan-Meier curve for overall survival stratified by an HLA pathway signature score (high vs. low). The TCGA-ACC cohort was dichotomized by the median of the sample scores, where each sample's score was the mean of the individual gene z-scores. (**B**) Kaplan-Meier curve stratified by four subgroups combining the steroid phenotype (HSP vs. LSP) and the HLA pathway signature score (high vs. low). The *p*-values in (**A**) and (**B**) are from the log-rank test. (**C**) Forest Plot showing the Hazard Ratios (HR) and 95% confidence intervals of a multivariate Cox model for overall survival, including the HLA pathway signature score, tumor stage, and steroid phenotype. The overall HR of the model is indicated at the bottom.

Table S1. Sample counts for each cancer type in the TCGA Pan-Cancer (PANCAN) dataset. The table lists the total number of tumor samples and the number of samples with available RNA-Seq data for each of the 33 cancer types analyzed. Abbreviations: ACC, Adrenocortical carcinoma; BLCA, Bladder Urothelial Carcinoma; BRCA, Breast invasive carcinoma; CESC, Cervical squamous cell carcinoma and endocervical adenocarcinoma; CHOL, Cholangiocarcinoma; COAD, Colon ade-

nocarcinoma; DLBC, Lymphoid Neoplasm Diffuse Large B-cell Lymphoma; ESCA, Esophageal carcinoma; GBM, Glioblastoma multiforme; HNSC, Head and Neck squamous cell carcinoma; KICH, Kidney Chromophobe; KIRC, Kidney renal clear cell carcinoma; KIRP, Kidney renal papillary cell carcinoma; LAML, Acute Myeloid Leukemia; LGG, Brain Lower Grade Glioma; LIHC, Liver hepatocellular carcinoma; LUAD, Lung adenocarcinoma; LUSC, Lung squamous cell carcinoma; MESO, Mesothelioma; OV, Ovarian serous cystadenocarcinoma; PAAD, Pancreatic adenocarcinoma; PCPG, Pheochromocytoma and Paraganglioma; PRAD, Prostate adenocarcinoma; READ, Rectum adenocarcinoma; SARC, Sarcoma; SKCM, Skin Cutaneous Melanoma; STAD, Stomach adenocarcinoma; TGCT, Testicular Germ Cell Tumors; THCA, Thyroid carcinoma; THYM, Thymoma; UCEC, Uterine Corpus Endometrial Carcinoma; UCS, Uterine Carcinosarcoma; UVM, Uveal Melanoma.

Cancer Type	Total Tumoral Samples	Tumoral Samples With RNA-Seq Data
ACC	92	79
BLCA	413	408
BRCA	1104	1102
CESC	309	306
CHOL	36	36
COAD	460	451
DLBC	48	48
ESCA	186	185
GBM	601	164
HNSC	530	522
KICH	66	66
KIRC	537	534
KIRP	292	291
LAML	200	173
LGG	529	525
LIHC	379	372
LUAD	521	517
LUSC	504	500
MESO	87	87
OV	600	307
PAAD	186	179
PCPG	184	184
PRAD	499	498
READ	167	160
SARC	265	263
SKCM	477	472
STAD	443	415
TGCT	139	139
THCA	515	513
THYM	124	120
UCEC	548	533
UCS	57	57
UVM	80	80
Total	11178	10286

Table S2. Clinicopathological characteristics of the ENSAT-ACC cohort stratified by C1A/C1B molecular subtypes. The table summarizes the characteristics of the 44 ACC cases. Values for categorical variables are presented as patient count (n), percentage of the subgroup total (%), and number

of deaths, in the format n (%) †deaths. Age is presented as mean ± standard deviation. Abbreviation: SD, Standard Deviation.

	C1A	C1B
**Total**	26 (100%) †17	18 (100%) †2
**Male**	6 (23%) †6	2 (11%) †2
**Female**	20 (77%) †11	16 (89%) †0
**Mean Age ± SD (Total)**	45.3 ± 19.2	44.8 ± 14.5
**Mean Age ± SD (Male)**	52.3 ± 17.0	46.0 ± 24.0
**Mean Age ± SD (Female)**	43.2 ± 19.7	44.6 ± 14.1
**Tumor Stage**		
Stage I	2 (8%) †1	2 (11%) †0
Stage II	11 (42%) †3	13 (72%) †0
Stage III	2 (8%) †2	0 (0%) †0
Stage IV	10 (38%) †10	3 (17%) †2
**Hormonal Secretion**		
Yes	26 (100%) †17	13 (72%) †2
No	0 (0%) †0	5 (28%) †0
